# TRIM29 promotes DNA virus infections by inhibiting innate immune response

**DOI:** 10.1038/s41467-017-00101-w

**Published:** 2017-10-16

**Authors:** Junji Xing, Ao Zhang, Hua Zhang, Jin Wang, Xian Chang Li, Mu-Sheng Zeng, Zhiqiang Zhang

**Affiliations:** 1 Immunobiology and Transplant Science Center, Houston Methodist Research Institute, Houston, TX 77030 USA; 2 State Key Laboratory of Oncology in South China, Sun Yat-sen University Cancer Center, Guangzhou, 510060 China; 3 Department of Surgery, Weill Cornell Medical College of Cornell University, New York, NY 10065 USA; 4 Collaborative Innovation Center for Cancer Medicine, Sun Yat-sen University Cancer Center, Guangzhou, 510060 China

## Abstract

Many double-stranded DNA viruses, such as Epstein-Barr virus, can establish persistent infection, but the underlying virus–host interactions remain poorly understood. Here we report that in human airway epithelial cells Epstein-Barr virus induces TRIM29, a member of the TRIM family of proteins, to inhibit innate immune activation. Knockdown of TRIM29 in airway epithelial cells enhances type I interferon production, and in human nasopharyngeal carcinoma cells results in almost complete Epstein-Barr virus clearance. TRIM29 is also highly induced by cytosolic double-stranded DNA in myeloid dendritic cells. *TRIM29* ^−/−^ mice have lower adenovirus titers in the lung, and are resistant to lethal herpes simplex virus-1 infection due to enhanced production of type I interferon. Mechanistically, TRIM29 induces K48-linked ubiquitination of Stimulator of interferon genes, a key adaptor in double-stranded DNA-sensing pathway, followed by its rapid degradation. These data demonstrate that Epstein-Barr virus and possible other double-stranded DNA viruses use TRIM29 to suppress local innate immunity, leading to the persistence of DNA virus infections.

## Abstract (web summary)

Proteins of the TRIM family have regulatory functions in immune signaling, often via ubiquitination of target proteins. Here, the authors show that TRIM29 is induced upon infection with DNA viruses, resulting in degradation of STING, decreased interferon signaling and increased pathogenicity in mice.

## Introduction

The airway epithelium and adjacent innate immune cells are responsible for orchestrating defense against inhaled viruses, bacteria, fungi, allergens, pollution, and other environmental insults. The human airway epithelium is a pseudostratified mucosal barrier, consisting of airway epithelial cells (AECs) and other cell types sitting at the interface between external environment and internal milieu of the respiratory tract^1, 2^. AECs are the first line of defense against invading pathogens including viruses in the respiratory tract by providing both a physical barrier and immunological functions. Under normal conditions, mucociliary and barrier functions of AECs help to limit the entry and the foster removal of pathogens^3^. However, it has become clear that AECs have a much more active role in the initiation of immune reactions. Under homeostatic conditions, AECs express many pattern recognition receptors including Toll-like receptors^4^, RIG-I^5^, C-type lectins^6^, and cyclic GMP–AMP synthase^7^ to rapidly detect and respond to pathogen-associated molecular patterns found in microbes or to damage-associated molecular patterns released upon tissue damage, cell death, or cellular stress. The activation of epithelial pattern recognition receptors on AECs leads to the release of type I interferon (IFN-I), cytokines, chemokines, and antimicrobial peptides that attract and activate innate and adaptive immune cells, which can function to clear the invading pathogens^8^, suggesting that AECs play critical roles in host defense against invading pathogens.

Although IFN-I was initially described based on their antiviral properties, it was quickly realized that these cytokines had antiproliferative and anticancer activities^9^. These observations ultimately led to the clinical use of IFN-I for the treatment of cancers such as melanoma, renal cell carcinoma, and chronic myelogenous leukemia and others^9^. Stimulator of interferon genes (STING) is the key adaptor in cytosolic DNA-sensing pathway^10^. STING pathway activation with antigen-presenting cells in the tumor microenvironment leads to production of IFN-I and the spontaneous generation of antitumor CD8^+^ T-cell responses^11^. STING agonists drive IFN-I production and are therapeutic in mouse tumor models^12–16^. Therefore, understanding the mechanisms of antitumor activities by IFN-I should enable improved clinical manipulation of the IFN-I system in cancer.

DNA virus Epstein-Barr virus (EBV) is a gamma herpesvirus and an oncogenic virus associated with a large number of human malignancies including nasopharyngeal carcinoma (NPC)^17^. EBV could infect both, B-memory lymphocytes and epithelial cells, causing a range of lymphoid and epithelial malignancies^17^. Indeed, EBV infection is found in almost all cases of NPC^18, 19^. Importantly, high levels of cell-free EBV DNA in blood are associated with poor survival of NPC patients^20^. Furthermore, high levels of EBV DNA were detected in NPC patients who relapsed after radiotherapy. By contrast, NPC patients with continuous clinical remission had low or undetectable levels of EBV DNA^19^. All these studies suggest a critical role for EBV infection in NPC. However, the mechanism of EBV in transformation in human epithelial malignancies remains unclear. Therefore, understanding the biology of EBV infection in human epithelial cells is crucial to the understanding of the pathogenic role of EBV in human epithelial malignancies including NPC.

In this study, we find that DNA viruses, including EBV, employ TRIM29, which is highly expressed in human AECs and double-stranded DNA (dsDNA)-induced in myeloid dendritic cells (mDCs), to suppress the host innate immune response. TRIM29 functions as a key negative regulator of the innate immune response to DNA viruses through targeting STING, leading to the persistence of DNA viruses.

## Results

### Human TRIM29 is specifically expressed in AECs and mDCs

**Fig. 1:**
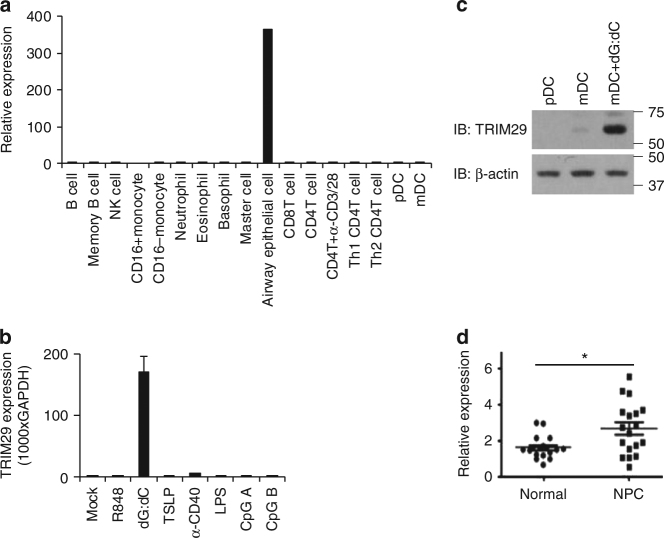
The expression of TRIM29 in human cells. **a**,**b** Human myeloid cells and lymphoid cells were purified from PBMCs using a cell sorter. Total RNA was isolated from these primary cells and primary airway epithelial cells induced or not **a** to chip hybridization and microarray, or **b** to real-time PCR. The profile of TRIM29 expression in different cells is indicated. The relative expression of TRIM29 was compared by plotting the values extracted from the gene expression database. A value <1 indicated the absence of gene expression. **c** Immunoblot analysis of TRIM29 and β-actin in plasmacytoid dendritic cells (pDCs), mDCs, and mDCs induced with poly(dG:dC) (dG:dC, 2.5 μg/ml). Data are representative of three independent experiments. **d** Total RNA was isolated from human healthy nasopharyngeal epithelium tissues (normal) and NPC tissues to chip hybridization and microarray. The relative expression of TRIM29 was compared by plotting the values extracted from the gene expression database. A value <1 indicated the absence of gene expression. *P < 0.0001 (unpaired *t*-test; mean and SD of three samples in **b**. The position of protein markers (shown in kDa) is indicated on the *right*

Microarray gene expression analysis showed that TRIM29 is specifically expressed in AECs (Fig. 1a). Furthermore, real-time PCR analysis showed that TRIM29 is also expressed in intestinal epithelial cells, but not in prostate epithelial cells or renal epithelial cells (Supplementary Fig. 1). Real-time PCR and immunoblot analysis showed that TRIM29 is highly induced by cytosolic dsDNA poly (dG:dC) in human primary mDCs from one random donor (Fig. 1b, c). Because nasopharynx AECs are the major target of EBV to induce NPC, we compared the TRIM29 expression levels in both human healthy nasopharyngeal epithelium tissues (normal) and NPC tissues, which are EBV-positive. Microarray gene expression analysis showed that the mRNA level of TRIM29 is indeed higher in NPC than in normal epithelium (Fig. 1d). These data showed that TRIM29 is highly expressed in AECs, dsDNA induced in mDCs and associated with EBV-positive NPCs.

### Human TRIM29 plays an important role in EBV-induced NPC

**Fig. 2:**
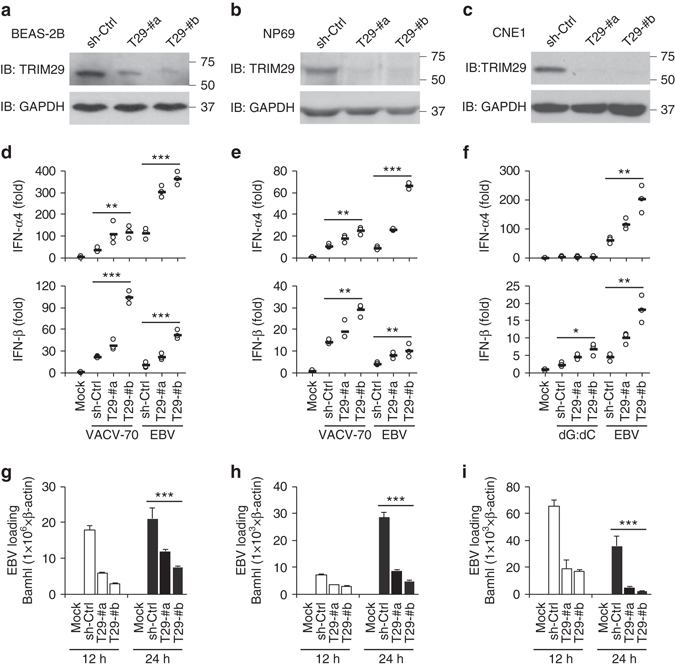
The function of TRIM29 in human airway epithelial cells and nasopharyngeal carcinoma. **a**–**c** Immunoblot analysis of TRIM29 in human bronchial epithelial cells BEAS-2B cells **a**, nasopharyngeal epithelial cells NP69 cells **b**, or nasopharyngeal carcinoma cells CNE1 cells **c** treated with control shRNA with scrambled sequence (sh-Ctrl), shRNA targeting mRNA encoding TRIM29 (two shRNAs: T29-#a and T29-#b). GAPDH serves as a loading control throughout. **d**–**f** Quantification of IFN-α4 and IFN-β mRNA expression in BEAS-2B cells **d**, NP69 cells **e**, or CNE1 cells **f** treated with scrambled shRNA (sh-Ctrl) and left unstimulated (Mock) or treated with shRNA as above and then stimulated for 6 h with VACV-70 (5 μg/ml) or poly(dG:dC) (dG:dC, 5 μg/ml) delivered by Lipofectamine 3000 or infection with EBV. Virus was used at a multiplicity of infection (MOI) of 5. **g**–**i** Quantification of EBV DNA loading in BEAS-2B cells **g**, NP69 cells **h**, or CNE1 cells (**i**) treated with scrambled shRNA (sh-Ctrl) and left unstimulated (Mock) or treated with shRNA as above and then infected with EBV for 12 or 24 h. Virus was used at a MOI of 5. Individual *circles* represent the value from independent experimental cells in each well; *small horizontal lines* indicate the average of triplicates. *P < 0.05, **P < 0.001, ***P < 0.0001 (unpaired *t*-test; mean and SD of three samples in **g**–**i**). Mock, cells without stimulation, or infection. The position of protein markers (shown in kDa) is indicated on the *right*. Data are representative of three independent experiments

To further investigate the role of TRIM29 in EBV-induced NPC, human healthy airway epithelial cell line BEAS-2B cells, EBV-negative nasopharyngeal epithelial cell line NP69 cells^21^, and human NPC cells CNE1 were employed for stable knockdown of TRIM29 expression through the use of short hairpin RNA (shRNA). Two distinct TRIM29-targeting shRNA constructs efficiently knocked down the expression of TRIM29 protein among BEAS-2B, NP69, or CNE1 cells (Fig. 2a–c). We stimulated the cells with dsDNA from VACV-70 or poly(dG:dC) delivered via lipofectamine 3000 for 6 h and measured the production of IFN-I in the cultured cells by quantitative real-time PCR. Knockdown of TRIM29 enhanced the productions of IFN-α4 and IFN-β by BEAS-2B cells, NP69 cells, and CNE1 cells by up to fourfold in response to intracellular VACV-70 or poly(dG:dC) (Fig. 2d–f). Because NPC is caused by EBV infection in human nasopharyngeal epithelial cells, we assessed the production of IFN-I by BEAS-2B cells, NP69 cells, and carcinoma cells CNE1 upon EBV infection. Knockdown of TRIM29 enhanced production of IFN-α4 and IFN-β after EBV infection by up to sevenfold as compared to the controls (Fig. 2d–f). We also measured viral titers of EBV in those cells by quantitative PCR, and found that the EBV viral load was lower in TRIM29 knockdown cells than in control cells (Fig. 2g–i). In line with these results, knockout (KO) of TRIM29 by CRISPR/Cas9 strategy also enhanced production of IFN-α and IFN-β in both, BEAS-2B and CNE1 cells, in response to intracellular VACV-70, poly(dG:dC) or EBV infection, and EBV viral loads were lower in TRIM29 KO cells than in control cells (Supplementary Fig. 2). These data showed that TRIM29 plays a critical role in suppressing IFN-I production and promoting EBV infection in NPC.

### TRIM29 regulates IFN-I production in dendritic cells

**Fig. 3:**
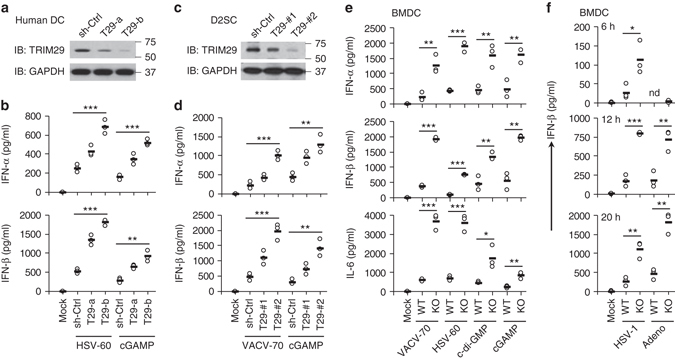
TRIM29 plays an important role in regulating IFN-I production in dendritic cells. **a** Immunoblot analysis of TRIM29 in human monocyte-derived dendritic cells (MonoDC) treated with control shRNA with scrambled sequence (sh-Ctrl), shRNA targeting mRNA encoding TRIM29 (two shRNAs: T29-a and T29-b). GAPDH serves as a loading control throughout. **b** ELISA of IFN-α and IFN-β in MonoDCs treated with scrambled shRNA (sh-Ctrl) and left unstimulated (Mock) or treated with shRNA as above and then stimulated for 16 h with dsDNA derived from herpes simplex virus type 1 (HSV-60, 2.5 μg/ml) or cGAMP (1.0 μg/ml) delivered by Lipofectamine 3000. **c** Immunoblot analysis of TRIM29 in mouse D2SC cells treated with control shRNA with scrambled sequence (sh-Ctrl), shRNA targeting mRNA encoding TRIM29 (two shRNAs: T29-#1 and T29-#2). GAPDH serves as a loading control throughout. **d** ELISA of IFN-α and IFN-β in D2SC cells treated with scrambled shRNA (sh-Ctrl) and left unstimulated (Mock) or treated with shRNA as above and then stimulated for 16 h with dsDNA from vaccinia virus (VACV-70, 2.5 μg/ml) or cGAMP (1.0 μg/ml) delivered by Lipofectamine 3000. **e** ELISA of IFN-α, IFN-β, and IL-6 in BMDCs from WT and *Trim29*-KO mice after 16 h of stimulation with dsDNA from vaccinia virus (VACV-70, 2.5 μg/ml), dsDNA from HSV-1 virus (HSV-60, 2.5 μg/ml), c-di-GMP (2.5 μg/ml), or cGAMP (1.0 μg/ml) delivered by Lipofectamine 3000. **f** ELISA of IFN-β in BMDCs from WT and *Trim29*-KO mice mock infected or infected with HSV-1 or adenovirus (adeno) at multiplicity of infection (MOI) of 5 for 6, 12, or 20. Each *symbol* represents the value from independent experimental cells in each well; *small horizontal lines* indicate the average of triplicates. Mock, cells without stimulation or infection. *P < 0.05, **P < 0.01, and ***P < 0.001 (unpaired *t*-test). The “nd” is defined as not detectable. The position of protein markers (shown in kDa) is indicated on the *right*. Data are representative of three independent experiments

Given that TRIM29 is also highly expressed in dsDNA-induced mDCs, we next determined the function of TRIM29 in both human dendritic cells and mouse splenic mDC cell line D2SC by stable knockdown of TRIM29 expression. Two distinct TRIM29-targeting shRNA constructs efficiently knocked down the expression of TRIM29 protein in both human dendritic cells and mouse dendritic cells (Fig. 3a, c). Knockdown of TRIM29 enhanced the production of IFN-α and IFN-β in human and mouse dendritic cells by up to fourfold in response to intracellular herpes simplex virus (HSV)-60, VACV-70, or cGAMP (Fig. 3b, d). These data suggested that dsDNA-induced TRIM29 negatively regulated the production of IFN-I in both human and mouse mDCs in response to cytosolic dsDNA. To further determine the role of TRIM29 in immune cells, we prepared bone marrow-derived dendritic cells (BMDCs) and bone marrow-derived macrophages (BMDMs) from wild-type mice (WT) and *Trim29*-KO mice. We then stimulated those BMDCs and BMDMs for 16 h with VACV-70, HSV-60, c-di-GMP (a ligand of STING), cGAMP, or zymosan. KO of TRIM29 enhanced production of IFN-I and pro-inflammatory cytokine interleukin (IL)-6 in BMDCs in response to VACV-70, HSV-60, c-di-GMP, or cGAMP twofold to fivefold (Fig. 3e). The kinetics of IFN-β expression in WT and KO BMDCs by dsDNA stimulation showed that IFN-β expression increased over time until reaching a maximum at around 12 h, and gradually decreased later (Supplementary Fig. 3). Similarly, compared with WT cells, *Trim29*-KO BMDMs produced twofold to threefold more IFN-α, IFN-β, TNF-α, and IL-6 in response to VACV-70, HSV-60, c-di-GMP, or cGAMP (Supplementary Fig. 4). By contrast, deletion of *Trim29* had no effect on the production of IFN-β and IL-6 in both BMDCs and BMDMs induced by zymosan (Supplementary Fig. 5). These data indicated a negative role for TRIM29 in sensing viral dsDNA, c-di-GMP, and cGAMP in both human and mouse immune cells. Infectious diseases as a result of DNA viruses, including EBV-induced NPC, are a major health concern worldwide. The common characterization of DNA viruses is to produce dsDNA when infecting the host cells^22^. IFN-I production by the host is the frontline antiviral defense strategy, and it is one of the main outcomes of the cytosolic sensing of DNA^22^. We therefore employed two classical DNA viruses, HSV-1 and adenovirus, to determine the role of TRIM29 in sensing DNA viruses in mouse mDCs. BMDCs from WT mice and *Trim29*KO mice were infected for 6, 12, or 20 h with HSV-1 and adenovirus. KO of TRIM29 enhanced production of IFN-β and IFN-α in BMDCs in response to HSV-1 or adenovirus infection twofold to fourfold at 6, 12, or 20 h post infection (Fig. 3f and Supplementary Fig. 6a). Similarly, compared with WT cells, *Trim29*-KO BMDMs produced twofold to fivefold more IFN-β and IFN-α in response to HSV-1 or adenovirus infection than did WT BMDMs at 6, 12, or 20 h post infection (Supplementary Figs. 6b and 7). Furthermore, *Trim29*-KO BMDCs and BMDMs produced twofold to threefold more IL-6 and TNF-α in response to HSV-1 or adenovirus infection than did WT BMDCs or BMDMs at 12 or 20 h post infection (Supplementary Fig. 8a–c). These data suggested a negative role for TRIM29 in regulating the innate immune response to DNA viruses in murine BMDCs and BMDMs.

### TRIM29 mediates host defense against viral infection *in vivo*

**Fig. 4:**
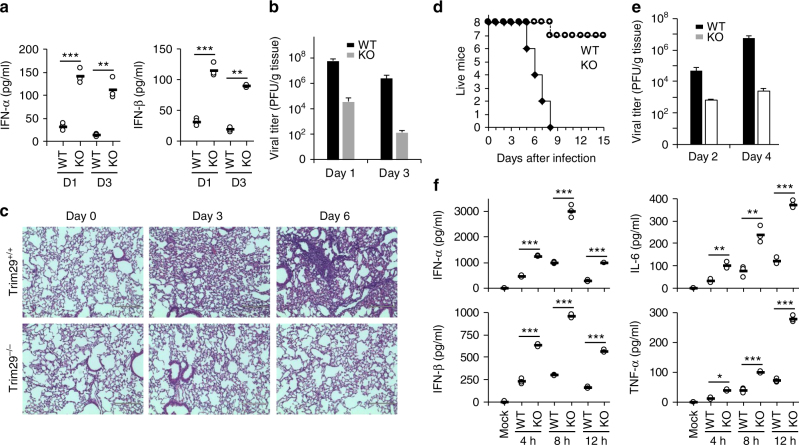
TRIM29 plays an important role in host defense against viral infection in vivo. **a** ELISA of IFN-α and IFN-β in BALF samples from WT and Trim29-KO mice (*n* = 3 per strain) on day 1 or day 3 of intranasal infection with adenovirus (1 × 10^8^ PFU per mouse). **b** Viral titers in homogenates of lung from WT and Trim29-KO mice (*n* = 3 per strain) on day 1 or day 3 of intranasal infection with adenovirus (1 × 10^8^ PFU per mouse). **c** Hematoxylin and eosin (H&E) staining of lung sections from *Trim29* ^+/+^ and *Trim29* ^−/−^ mice intranasally infected with adenovirus (1 × 10^8^ PFU per mouse) on day 0, day 3, or day 6. *Scale bars* represent 400 µm. **d** Survival of WT and *Trim29*-KO mice (*n* = 8 per strain) after intravenous injection of HSV-1 (2 × 10^7^ PFU per mouse). **e** Viral titers in homogenates of brains from WT and *Trim29*-KO mice (*n* = 3 per strain) after intravenous injection of HSV-1 (2 × 10^7^ PFU per mouse). **f** ELISA of IFN-α, IFN-β, TNF-α, and IL-6 in the serum obtained from WT and *Trim29*-KO mice (*n* = 3 per strain) at various times (*horizontal axes*) after intravenous injection of HSV-1 (2 × 10^7^ PFU per mouse). Each symbol represents the value from independent experimental cells in each well; *small horizontal lines* indicate the average of triplicates. *P < 0.05, **P < 0.01, and ***P < 0.001 (unpaired *t*-test). Data are representative of three experiments (mean and SD of three samples in **b**, **e**)

We next evaluated the importance of TRIM29 in host defense against DNA viral infection *in vivo*. We first intranasally infected both WT mice and *Trim29*-KO mice with adenovirus, which is transmitted by nasal route in nature. *Trim29*-KO mice produced threefold to fivefold more IFN-I in the bronchoalveolar lavage fluid (BALF) than did WT mice at day 1 or day 3 post infection (Fig. 4a). Also, there were onefold to threefold more TNF-α and IL-6 detected in BALF from *Trim29*-KO mice, as compared to that in WT mice on day 1 or day 3 post infection (Supplementary Fig. 9). We also measured viral titers of adenovirus in lung homogenates by plaque-forming assay after infection, and found that the adenovirus load was reduced by 10,000-fold in *Trim29*-KO mice (Fig. 4b). Furthermore, lung histopathology revealed much-reduced edema, alveolar hemorrhage, alveolar wall thickness, and neutrophil infiltration in *Trim29*-knockout mice as compared to the lung pathology in WT mice after viral infection (Fig. 4c). These data indicate that TRIM29 inhibits innate immune activation, especially the production of IFN-I in respiratory tract, thus allowing the virus to escape host immune attacks.

We also challenged WT mice and *Trim29*-KO mice intravenously with HSV-1 virus, and monitored survival over time. Relative to WT controls, *Trim29*-KO mice had significantly higher survival rates (Fig. 4d). Furthermore, we measured viral titers of HSV-1 in the brain, liver, and spleen by plaque assay on day 2 and day 4 after infection. We detected significantly less HSV-1 virus in *Trim29*-KO mice than in WT mice (Fig. 4e and Supplementary Fig. 10). Additionally, *Trim29*-KO mice had higher concentrations of IFN-I, IL-6, and TNF-α in the serum than did their WT littermates after infection with HSV-1 (Fig. 4f). These data suggested that TRIM29 negatively controlled DNA virus-triggered signaling and *Trim29* deficiency could protect mice from DNA virus challenge.

### TRIM29 inhibits the expression of STING

**Fig. 5:**
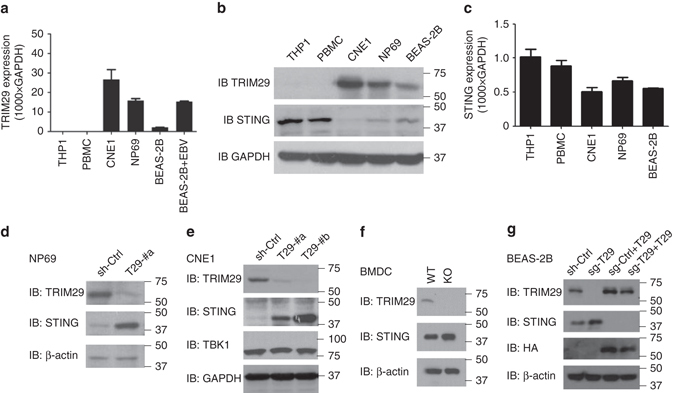
TRIM29 inhibits the expression of STING. **a** Quantification of TRIM29 mRNA expression in human THP-1 cells, PBMCs, CNE1 cells, NP69 cells, BEAS-2B cells, and BEAS-2B cells with EBV infection at MOI of 5. **b** Immunoblot analysis of TRIM29 and STING expression in human THP-1 cells, PBMC, CNE1 cells, NP69 cells, and BEAS-2B cells. **c** Quantification of STING mRNA expression in human THP-1 cells, PBMCs, CNE1 cells, NP69 cells, and BEAS-2B cells. **d**–**f** Immunoblot analysis of TRIM29, STING, and TBK1 expression in human healthy NP69 cells **d**, human NPC cell line CNE1 cells **e** treated with control shRNA with scrambled sequence (sh-Ctrl), shRNA targeting mRNA encoding TRIM29 (two shRNAs: T29-#a and T29-#b), or BMDCs **f** isolated from WT and *Trim29* ^−/−^ KO mice. **g** Immunoblot analysis of TRIM29 and STING expression in human bronchial epithelial BEAS-2B cells treated by CRISPR vector control (sg-Ctrl) or sgRNA targeting 5′ UTR region of TRIM29 (sg-T29) with or without overexpression of HA-tagged TRIM29. GAPDH and β-actin serve as loading control. The position of protein markers (shown in kDa) is indicated on the *right*. Data are representative of three experiments (mean and SD of three samples in **a**, **c**)

The previous data showed that TRIM29 was highly expressed in human AECs and NPC (Fig. 1). We next determined the TRIM29 expression in different types of human cells with or without EBV infection by quantitative real-time PCR. TRIM29 mRNA was undetectable in both human THP1 and peripheral blood mononuclear cells (PBMCs; Fig. 5a). In contrast, TRIM29 was highly expressed in human epithelial BEAS-2B cells, non-neoplastic epithelial NP69 cells, and NPC CNE1 cells (Fig. 5a). Importantly, TRIM29 was highly induced in BEAS-2B cells after EBV infection (Fig. 5a). To further investigate how TRIM29 regulates dsDNA or DNA virus-induced signaling events, we identified TRIM29-interacting proteins by immunoprecipitation with antibody to TRIM29 (anti-TRIM29) in the mouse mDC line D2SC, followed by protein sequencing by liquid chromatography–mass spectrometry. We identified STING among the group of TRIM29-interacting proteins (Supplementary Table 1). KO of STING dramatically reduced the productions of IFN-α and IFN-β by CNE1 cells after EBV infection (Supplementary Fig. 11), suggesting that STING is essential for EBV-triggered IFN-I production in human NPC. We therefore determined the protein levels of both TRIM29 and STING in those above cells. Indeed, TRIM29 protein was highly expressed in CNE1, NP69, and BEAS cells, but not in THP1 cells and PBMCs (Fig. 5b). Interestingly, the highly expressed TRIM29 protein in cells could reduce STING expression, while STING was highly expressed in THP1 and PBMC with low TRIM29 expression (Fig. 5b). Furthermore, real-time PCR analysis showed that TRIM29 plays little role in affecting STING mRNA expression among those cells (Fig. 5c). To further confirm the relationship of TRIM29 and STING in EBV induced NPC, NP69 cells, and CNE1 cells were employed for stable knockdown of TRIM29 expression through the use of shRNA. Knockdown of TRIM29 expression could rescue the high expression of STING in both NP69 cells (Fig. 5d) and CNE1 cells (Fig. 5e). By contrast, knockdown of TRIM29 did not affect the expression of TBK1 (Fig. 5e), a key adaptor in DNA-sensing pathway. Compared with BMDCs from WT mice, the protein level of STING is increased in BMDCs from *Trim29*-KO mice(Fig. 5f). Importantly, the reconstitution of TRIM29 in TRIM29-KO BEAS-2B cells could rescue the TRIM29 expression and reduce the protein level of STING (Fig. 5g). These data suggested that TRIM29 inhibited the expression of STING in AECs, BMDCs, and especially in NPC.

### TRIM29 binds and colocalizes with STING

**Fig. 6:**
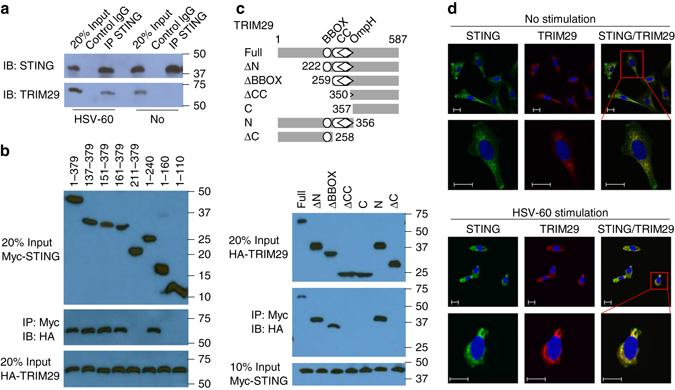
TRIM29 binds and colocalizes with STING in the cytosol. **a** Immunoblot analysis of endogenous proteins of TRIM29 and STING precipitated with anti-STING or immunoglobulin G (IgG; control) from whole-cell lysates of BMDCs from WT mice stimulated without (No) or with HSV-60 (2.5 μg/ml) for 8 h, and then with MG132 treatment for 3 h. Input, 20% of the BMDCs lysate. **b** Full-length STING and serial truncations of STING with deletion of various domains (*top*). Immunoblot analysis of purified HA-tagged TRIM29 with anti-HA (*bottom blot*), and immunoblot analysis of purified HA-tagged full-length TRIM29 with anti-HA (*middle blot*) or purified Myc-tagged full-length STING and STING truncation mutants alone with anti-Myc (*top blot*) after incubation with Myc-tagged full-length STING and STING truncation mutants and immunoprecipitation with anti-Myc (*top* and *middle blots*). **c** Full-length TRIM29 and serial truncations of TRIM29 with deletion (Δ) of various domains (*left margin*); numbers at ends indicate amino-acid positions (*top*). *Below*, immunoblot analysis of purified Myc-tagged STING with anti-Myc (*bottom blot*), and immunoblot analysis (with anti-HA) of purified HA-tagged full-length TRIM29 and TRIM29 truncation mutants alone (*top blot*) or after incubation with Myc-tagged STING and immunoprecipitation with anti-Myc (*middle blot*). **d** Colocalization of endogenous TRIM29 and STING in BEAS-2B cells. Confocal microscopy of BEAS-2B cells without stimulation, or stimulated with dsDNA HSV-60 for 4 h. STING was stained with Rabbit anti-STING polyclonal antibody (Cat: 19851-1-AP, Proteintech), followed by Alexa Fluor 488 goat anti-rabbit secondary antibody (*green*), while TRIM29 was stained with mouse anti-TRIM29 monoclonal antibody (sc-166707, Santa Cruz), followed by Alexa Fluor 594 goat anti-mouse secondary antibody (*red*). DAPI (4′,6-diamidino-2-phenylindole) served as the nuclei marker (*blue*). *Scale bars* represent 20 µm. The position of protein markers (shown in kDa) is indicated on the *right*. Data are representative of three independent experiments

STING is a key adaptor molecule with antiviral activity in the DNA-sensing pathway. Our IP-MS (immunoprecipitation–mass spectrometry) data also showed that STING was among the group of TRIM29-interacting proteins (Supplementary Table 1). We next determined the interaction between TRIM29 and STING in BMDCs at an endogenous protein level. Anti-STING antibody, but not control IgG, could pull down STING and TRIM29 in BMDCs activated by cytosolic dsDNA of HSV-60. By contrast, anti-STING antibody can not pull down TRIM29 without dsDNA stimulation, suggesting that there was indeed interaction between TRIM29 and STING in BMDCs under condition of cytosolic dsDNA stimulation (Fig. 6a). To further map the binding sites between STING and TRIM29, we analyzed the interactions among Myc-tagged recombinant STING and truncation mutants of STING and hemagglutinin (HA)-tagged recombinant full-length TRIM29 and truncation mutants of TRIM29. As results, both recombinant full-length STING and its truncation mutants, except for aa211–379, 1–160, or 1–110 of STING, interacted with TRIM29, suggesting that the amino acids 161–240 in c-di-GMP-binding domain of STING bound TRIM29 (Fig. 6b). Additionally, both recombinant full-length TRIM29 and the OmpH domain of TRIM29 bound STING (Fig. 6c). Next, we expressed Myc-tagged STING together with HA-tagged full-length TRIM29 (T29) or TRIM29 with truncation of the ∆C domain (T29-∆C, which lacks the STING-binding site) in HEK293T cells to determine their subcellular localization. Immunofluorescence imaging showed STING colocalized with the full-length TRIM29, but not the truncation lacking the STING-binding site (Supplementary Fig. 12). Furthermore, immunofluorescence of TRIM29 and STING showed that endogenous TRIM29 colocalized with endogenous STING in the cytosol, and TRIM29 translocated to the perinuclear region in BEAS-2B cells after dsDNA stimulation (Fig. 6d). Collectively, these data suggested that TRIM29 could bind and colocalize with STING in the cytosol.

### TRIM29 ubiquitinates and degrades STING

**Fig. 7:**
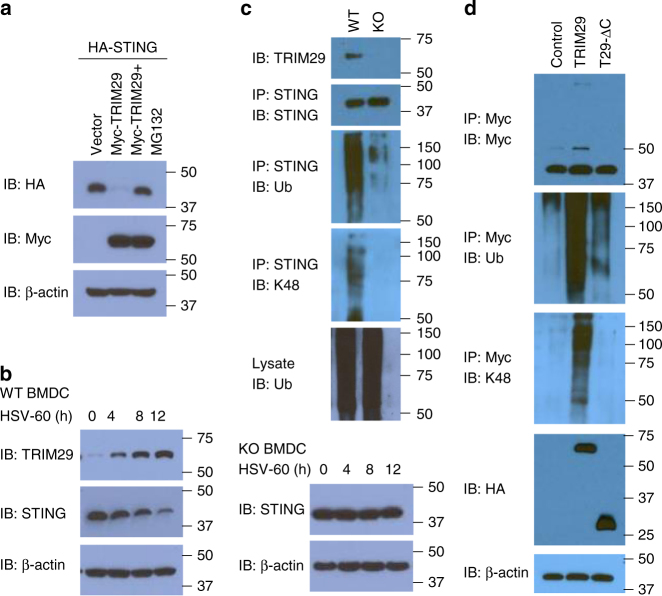
TRIM29 induces ubiquitination of STING by K48-linkage. **a** Immunoblot analysis of HA-tagged STING (*top blot*), Myc-tagged TRIM29 (*middle blot*), and β-actin (*bottom blot*) in HEK293T cells co-transfected with expression vector for HA-tagged STING and with empty vector or expression vector for Myc-tagged TRIM29, with or without treatment of 5 μM MG132 (*above lanes*). **b** Immunoblot analysis of TRIM29 (*top blot*), STING (*middle blot*), and β-actin (*bottom blot*) in both WT and TRIM29 KO BMDCs stimulated for various times (*above lanes*) with HSV-60 (2.5 μg/ml). **c** Immunoblot analysis of TRIM29 in WT and KO BMDCs (*top*), and of the abundance (*second blot*), total ubiquitination (*third blot for IP, bottom blot for lysate*), and K48-mediated ubiquitination (*fourth blot*) of STING in those cells, stimulated for 4 h with HSV-60 (2.5 μg/ml), assessed after immunoprecipitation with anti-STING. **d** Immunoblot analysis (with anti-Myc) of the abundance (*top*), total ubiquitination (*second blot*), and K48-linked ubiquitination (*third blot*) of Myc-tagged STING in HEK293T cells transfected with empty vector or expression vector for HA-tagged TRIM29, truncation T29-∆C (losing binding site of STING), and stimulated for 4 h with HSV-60 (2.5 μg/ml), assessed after immunoprecipitation with anti-Myc; immunoblot analysis of whole-cell lysates with anti-HA (*fourth blot*) and anti-β-actin (*bottom*). The position of protein markers (shown in kDa) is indicated on the *right*. Data are representative of three experiments

Because TRIM29 is an E3 ubiquitin ligase, we next determined whether STING is the ubiquitination target of TRIM29. We co-expressed HA-STING with Myc-TRIM29 or vector control in HEK293T cells with or without treatment of MG132 and analyzed the expression of STING and TRIM29. As results, TRIM29 could significantly degrade STING, compared with the vector control (Fig. 7a). Additionally, the treatment of MG132 could rescue the expression of STING (Fig. 7a), suggesting that STING is indeed the ubiquitination target of TRIM29 in the proteasome manner. To determine whether the expression of both TRIM29 and STING was regulated by stimulation with dsDNA, we stimulated cells with HSV-60 for 0, 4, 8, or 12 h and then measured the protein levels of TRIM29 and STING in BMDCs. TRIM29 was upregulated in WT BMDCs after stimulation with HSV-60, while STING was downregulated after incubation with HSV-60 due to the degradation by TRIM29 (Fig. 7b). By contrast, STING was constitutively expressed in BMDCs from *Trim29*-KO mice (Fig. 7b). Collectively, these data indicated that dsDNA induced TRIM29 could result in STING protein degradation.

To determine whether TRIM29 was responsible for the ubiquitination of STING ex vivo, we stimulated BMDCs from WT mice or *Trim29*-KO mice for 4 h with HSV-60. Cell lysates were prepared and analyzed for the ubiquitination of STING. STING was modified via K48-mediated ubiquitination in BMDCs from WT mice but not *Trim29*-KO mice (Fig. 7c). To investigate whether the ubiquitination of STING was dependent on the binding site of TRIM29 with STING, we transfected the HEK293T cells to coexpress Myc-tagged STING and HA-tagged full-length TRIM29, or truncated TRIM29 lacking the binding site of STING (T29-∆C). We stimulated the cells for 4 h with dsDNA, and then prepared cell lysates and incubated them for 5 min at 90 °C with 1% SDS (sodium dodecyl sulfate) to disrupt protein–protein interactions, followed by immunoprecipitation of Myc-tagged STING. Immunoblot analysis of HA or ubiquitin demonstrated that the ubiquitination of STING was strongly enhanced by overexpression of TRIM29 but not by overexpression of T29-∆C (Fig. 7d). Immunoblot analysis of K48-linked ubiquitin further demonstrated that TRIM29 induced ubiquitination of STING by K48-mediated linkage (Fig. 7d). Together, these data indicated that TRIM29 targeted STING and induced its ubiquitination for protein degradation by K48-linkage.

### TRIM29 induces STING ubiquitination at its lys370 site

**Fig. 8:**
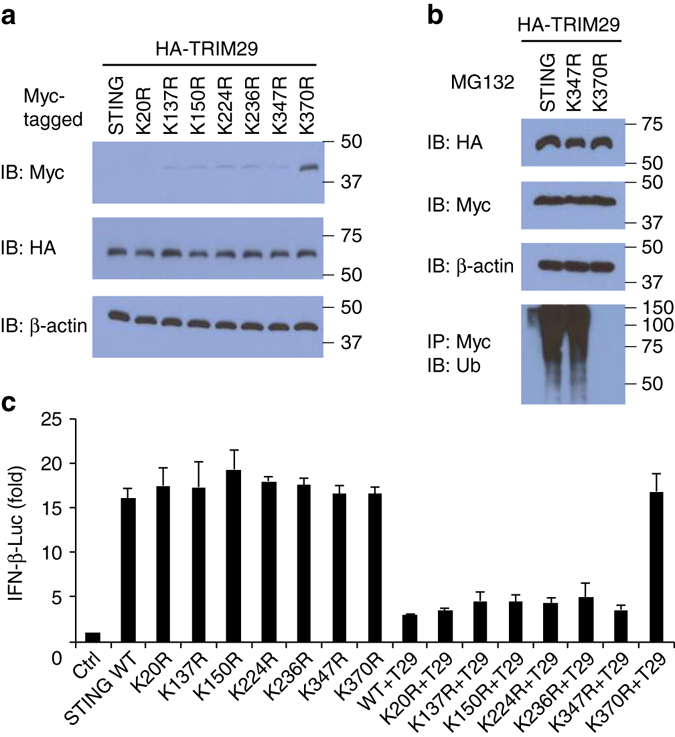
TRIM29 induces STING degradation upon ubiquitination at the Lys370 site. **a** Immunoblot analysis of Myc-tagged STING and its mutations (*top blot*), HA-tagged TRIM29 (*middle blot*), and β-actin (*bottom blot*) in HEK293T cells co-transfected with expression vector for HA-tagged TRIM29 and expression vectors for Myc-tagged WT full-length STING and its mutations. **b** Immunoblot analysis of Myc-tagged STING and its mutations (*second blot*), HA-tagged TRIM29 (*top blot*), and β-actin (*third blot*) and total ubiquitination (*bottom blot*) in HEK293T cells co-transfected with expression vector for Myc-tagged STING or its mutations (K347R or K370R) and expression vector for HA-tagged TRIM29, with treatment of 5 μM MG132 (*above lanes*). **c** Activation of the *Ifnb* promoter in human HeLa cells transfected with an *Ifnb* luciferase reporter, plus expression vector (each 100 ng) for WT STING or various STING mutants alone or expression vector for TRIM29 plus WT STING or STING mutants with stimulation of dsDNA from vaccinia virus (2.5 μg/ml) for 8 h; results are presented relative to those of renilla luciferase (co-transfected as an internal control). The position of protein markers (shown in kDa) is indicated on the *right*. Data are representative of three independent experiments (mean and SD of three samples in **c**)

STING contains nine lysine residues. Eight of these (Lys20, Lys137, Lys150, Lys224, Lys236, Lys347, and Lys370) were predicted to be possible ubiquitination sites with high confidence scores by the UbPred program that predicts such sites or reported previously^23^ (Supplementary Table 2). To determine the STING-ubiquitination sites, we replaced each of those STING lysine residues noted above individually with arginine. We coexpressed HA-tagged TRIM29 with Myc-tagged WT STING and its mutants (K20R, K137R, K150R, K224R, K236R, K347R, and K370R) in HEK293T cells and detected their expressions. As results, TRIM29 expressed similarly and could degrade the WT STING and its mutants (K20R, K137R, K150R, K224R, K236R, and K347R), but not STING mutant K370R (Fig. 8a). To further determine whether the ubiquitination of STING by TRIM29 was affected by the mutation of STING, we transfected the HEK293T cells to express Myc-tagged STING or its mutants (K347R or K370R) and HA-tagged full-length TRIM29 with treatment of MG132. Cells were stimulated for 4 h with dsDNA. Cell lysates were prepared and followed by immunoprecipitation of Myc-tagged STING. Immunoblot analysis demonstrated that both Myc-tagged STING or its mutants (K347R or K370R) and HA-tagged full-length TRIM29 expressed similarly. As expected, the ubiquitination of STING or its mutant K347R was strongly enhanced by overexpression of TRIM29 (Fig. 8b). However, the ubiquitination of STING mutant K370R by TRIM29 was absent (Fig. 8b).

Through the use of a luciferase reporter assay of the *Ifnb* promoter, established in the human HeLa cells, we found that overexpression of STING enhanced the activity of the *Ifnb* promoter in response to stimulation with dsDNA from vaccinia virus (Fig. 8c), but not in HeLa cells without stimulation (Supplementary Fig. 13). Overexpression of mutant STING with the K20R, K137R, K150R, K224R, K236R, K347R, or K370R substitution could still enhance the activity of the *Ifnb* promoter after stimulation with dsDNA (Fig. 8c). In addition, overexpression of TRIM29 inhibited the activity of the *Ifnb* promoter in HEK293T cells expressing WT STING or mutant STING with the K20R, K137R, K150R, K224R, K236R, or K347R substitution. However, overexpression of TRIM29 did not inhibit the activity of the *Ifnb* promoter in HEK293T cells expressing mutant STING with the K370R substitution (Fig. 8c). These data indicated that Lys370 was a critical site for TRIM29-mediated ubiquitination and regulation of STING.

## Discussion

Worldwide, one-fifth of cancers in the human population are associated with viral infections^24^. EBV is a DNA virus associated with NPC. NPC is a cancer arising from the nasopharynx epithelium. About 86,500 cases of NPC were reported worldwide in 2012. NPC is a relatively frequent disease in eastern countries, reaching an annual incidence rate of 40–50/100,000^25^. EBV is a human cancer-associated dsDNA virus that infects >90% of the global population^26^. Increasing evidence shows that TRIM29 is involved in a variety of cancers. Microarray analysis indicates that TRIM29 is overexpressed in the lung, pancreatic, gastric, bladder, colorectal, ovarian, and endometrial cancers, as well as plasma cell myeloma^27–32^. It is shown that upregulated TRIM29 promotes proliferation and metastasis of NPC^33, 34^. However, the biological function and clinical significance of highly expressed TRIM29 in NPC remain unclear. In this study, we found that human AECs selectively express TRIM29 and its expression can be further induced by EBV infection. Expression of TRIM29 then degrades STING, which inhibits the production of IFN-I and suppresses local innate immune responses. We provide evidence that TRIM29 interacts with STING and induces its ubiquitination at Lys370 site by K48-mediated linkage for protein degradation. IFN-I is a cytokine with a long record of use in clinical oncology^35^, and IFN-I has been shown to kill NPC directly^36^. It is reported that TRIM29 controls the innate immunity against respiratory viral and bacterial infections in the respiratory tract^37^. TRIM29 itself has also been shown to increase aggressiveness of certain cancers including gastric cancer^38^ and pancreatic cancer^39^, and the protein of TRIM29 induces progression of NPC^33^. Knockdown of TRIM29 in AECs eliminates EBV infection, a virus that is known in the development of NPC. Deficiency of TRIM29 also enhanced the expression of STING, which plays a critical role in antitumor immunity and cancer immunotherapeutics^11–16, 40^. All those studies suggest that TRIM29 is a critical target for NPC therapy. Further studies have shown that TRIM29 was highly expressed in dsDNA-induced mDCs and negatively regulate IFN-I and proinflammatory cytokines productions in response to cytoplasmic dsDNA, STING ligands or DNA viral infection. Importantly, *Trim29* ^−/−^ mice infected by HSV-1 or adenovirus exhibited greatly reduced morbidity, enhanced IFN-I and cytokine production, and reduced viral load.

STING exerts pivotal roles in cytosolic nucleic acid-triggered innate immune response. Activation of the STING pathway is a central innate immune-sensing mechanism that leads to IFN-I production in the tumor microenvironment. As tumors with a IFN-I signature correlate with infiltration of CD8^+^ T cells, the use of intratumoral STING agonists holds promise as a cancer therapeutic^40^. Until now, multiple mechanisms are involved in modulating STING-mediated signal transduction for generating an appropriate immune response. One such mechanism is K48-mediated ubiquitination to degrade STING. It is reported that the E3 ubiquitin ligase RNF5 induces the K48-linked polyubiquitination of STING at Lys150 for subsequent degradation of STING, thereby downregulating the RNA virus-induced IFN-I response^41^. However, RNF5 is not expressed in human nasal respiratory epithelium where EBV majorly infected in NPC (Supplementary Fig. 14). A recent study has identified TRIM30a as a negative-feedback regulator of the DNA virus-triggered response by targeting STING for degradation via K48-linked ubiquitination^42^. However, humans do not possess TRIM30a^43^. To the best of our knowledge, TRIM29 represents the first E3 ligase to induce STING degradation in human cells. It is reported that TRIM29 induces ubiquitination and degradation of NEMO using an atypical B-box domain as E3 ligase activity^37^. Therefore, TRIM29 may use E3 ligase activity for degrading STING in the same manner. The second mechanism is K63-mediated or K27-mediated ubiquitination or sumoylation of STING for signaling. It has been shown that either TRIM family protein TRIM56 or TRIM32 promotes STING-dependent innate immune responses by inducing the K63-linked polyubiquitination of STING^44, 45^. It is found that ER (endoplasmic reticulum)-resident autocrine motility factor receptor and the insulin-induced gene 1 (INSIG1) protein complex mediate the K27-linked polyubiquitination of STING and thereby recruit TBK1 for activating efficient downstream signaling transduction^46^. TRIM38 has been shown to regulate the sumoylation of STING^47^. The third mechanism is to regulate the duration and intensity of STING signaling by phosphorylation. It is reported that cGAMP induces the phosphorylation of STING at S366 by Unc51-like autophagy activating kinase 1, which inactivates STING and then prevents sustained innate immune signaling^48^. Further studies suggest that the phosphorylation of STING at S366 is required for the direct binding of STING to a positively charged surface of IRF3 and activation of downstream signaling, because the S366A and S366D mutations of STING are inactive^49, 50^. The last mechanism is to regulate the stability of the STING–TBK1 complex. It was reported that NLRC3^51^, ZDHHC1^52^, and iRhom2^53^ mediate the positive regulation of STING signaling to stabilize the STING complex. Although Lys20, Lys 150, Lys224, and Lys 236 have been identified as ubiquitination sites of STING^23^, we herein demonstrated that TRIM29 induced the ubiquitination of STING at Lys370 by K48-mediated linkage for protein degradation. Lys370 was identified as the ubiquitination site of STING.

The activation of sensors and adaptors to recognize viral nucleic acids by immune cells is critical for the elimination of invading microorganisms^54^. However, uncontrolled production of IFN-I and cytokines induced by DNA and RNA may lead to autoimmune and inflammatory diseases, such as systemic lupus erythematosus. The identification of TRIM29 as a negative regulator of STING in DNA-sensing will have important implications for the understanding of not only antiviral innate immunity but also the pathogenesis of EBV-induced NPC as well as human autoimmune diseases.

## Methods

### Mice

*Trim29* KO (*Trim29* ^−/−^) mice were from the European Mouse Mutant Archive (EMMA, EM:07120). Primary bone marrow was collected from WT and *Trim29* ^−/−^ mice for inducing mature BMDCs and BMDMs. All animals were maintained in a specific pathogen-free facility at Houston Methodist Research Institute in Houston, Texas. Animal use and care were approved by the Houston Methodist Animal Care Committee, in accordance with institutional animal care and use committee guidelines.

### Reagents

The dsDNA from vaccinia virus (VACV-70, Catalog: tlrl-vav70n), dsDNA from herpes simplex virus type 1 (HSV-60, Catalog: tlrl-hsv60n), poly(dG:dC) (Catalog: tlrl-pgcc), c-di-GMP (Catalog: tlrl-nacdg23), cGAMP (Catalog: tlrl-nacga23), R848 (TLR7/8 agonist, Catalog: tlrl-r848), CpG A (Catalog: tlrl-2216), CpG B (Catalog: tlrl-2006), and zymosan (Catalog: tlrl-zyn) were from Invivogen. Lipofectamine 3000 was from Invitrogen. The proteasome inhibitor MG132 was from Sigma. The following antibodies were used for immunoprecipitation: anti-STING (1:100; clone D2P2F; 13647; Cell Signaling Technology) and anti-TRIM29 (1:100; A301-210A; Bethyl). The following antibodies were used for immunoblot analysis: anti-STING (1:100; clone D2P2F; 13647; Cell Signaling Technology), anti-TRIM29 (1:1000; A301-210A; Bethyl), anti-TRIM29 (1:1000; sc-33151; H-300; Santa Cruz), anti-TBK1 (1:1000; 3013 S, Cell Signaling Technology), anti-ubiquitin (1:1000; sc-8017; Santa Cruz), K63-specific anti-ubiquitin (1:1000; 05-1313; Millipore), K48-specific anti-ubiquitin (1:1000; 05-1307; Millipore), anti-GAPDH (1:10,000; clone GAPDH-71.1, G9295; Sigma), anti-HA (1:5000; clone HA-7, H6533; Sigma), anti-β-actin (1:20,000; clone AC-15, A3854; Sigma), anti-Myc (1:5000; ab1326; Abcam), and anti-GST (1:1000; ab58626; Abcam). The following antibodies were used for confocal microscopy: Alexa Fluor 555-anti-Myc (1:50; clone 9B11, 3756; Cell Signaling), Alexa Fluor 488-anti-HA (1:50; clone 6E2, 2350; Cell Signaling), rabbit anti-STING polyclonal antibody (1:500; 19851-1-AP; Proteintech), mouse anti-TRIM29 monoclonal antibody (1:500; sc-166707; Santa Cruz), Alexa Fluor 488 goat anti-rabbit secondary antibody (A-11034; Thermo Fisher Scientific), and Alexa Fluor 594 goat anti-mouse secondary antibody(A-11005; Thermo Fisher Scientific). Anti-HA and anti-Myc beads were from Sigma. Lentiviral vectors for shRNA were as follows (all from Open Biosystems): mouse TRIM29 (clone TRCN0000241302 (TRIM29-#1) and clone TRCN0000241305 (TRIM29-#2)), human TRIM29 (clone TRCN0000016348 (TRIM29-#a), and clone TRCN0000016352 (TRIM29-#b)). The IFN-β ELISA kit was from PBL InterferonSource. The IFN-α, IL-6, and TNF-α ELISA kits were from eBioscience. The Dual-Luciferase Reporter Assay System (E1910) was from Promega.

### Cells culture and lentiviral infection

The institutional review board for human research at the Houston Methodist Research Institute approved this study. Human monocyte-derived dendritic cells were isolated from buffy coats of individual healthy donors and maintained in RPMI-1640 medium supplemented with 10% heat-inactivated fetal calf serum (FCS) and 1% penicillin–streptomycin (Invitrogen-Gibco) as previously described^55, 56^. D2SC cells^60^ were maintained in Iscove’s modified Dulbecco’s medium containing 10% heat-inactivated FCS and 1% penicillin–streptomycin (Invitrogen-Gibco). BEAS-2B cells (ATCC CRL-9609) were grown in BEBM media (Lonza, Cat. CC3171), with an added enhancement bullet kit (Lonza, Cat. CC3170), 1% penicillin–streptomycin (Invitrogen-Gibco) and Fungizone (5 ml) to create basal epithelial grown media. NP69 cell line^57^ was cultured in keratinocyte serum-free medium (Invitrogen) supplemented with 5% heat-inactivated FCS, 1% penicillin–streptomycin (Invitrogen-Gibco), 25 μg/ml bovine pituitary extract, and 0.2 ng/ml recombinant epidermal growth factor. The NPC cell line CNE1^58^ was cultured in RPMI-1640 medium supplemented with 5% heat-inactivated FCS and 1% penicillin–streptomycin (Invitrogen-Gibco). All cell lines were tested without mycoplasma contamination. MonoDC, D2SC, BEAS-2B, NP69, and CNE1 cells were infected with a pLKO.1 lentiviral vector carrying a scrambled shRNA (RHS6848, Open Biosystems) or target gene sequences (Open Biosystems) as described in our previous studies^59, 60^. After 24 h of culture, cells were selected by the addition of puromycin (2 ng/ml) to the medium. Cells were stimulated for 6 h with HSV-60 (5 μg/ml), VACV-70 (5 μg/ml), poly(dG:dC; 5 μg/ml), or cGAMP (1.0 μg/ml) delivered by Lipofectamine 3000. The knockdown efficiency was detected with immunoblot analysis.

### KO of TRIM29 or STING by using CRISPR/Cas9 vector

We employed the lentiviral expressing CRISPR–Cas9 vector (lentiCRISPR v2, Addgene plasmid #52961) to generate TRIM29 or STING KO cell lines. Specifically, this one vector system expresses the sgRNA, Cas9 protein, and puromycin resistance gene from one virus. The TRIM29 and STING sgRNA was designed using the Zhang lab software available at http://crispr.mit.edu. The gene-specific sgRNA sequences cloned were: TRIM29 (forward, 5′-CACCGCCACTCATTGCCCGCGAAC-3′; reverse, 5′-AAACGTTCGCGGGCAATGAGTGGC-3′). STING (forward, 5′-CACCGGGATGTTCAGTGCCTGCGAG-3′; reverse, 5′-AAACCTCGCAGGCACTGAACATCCC-3′). Empty lentiCRISPR v2 vector was used as a control. Correct cloning was confirmed by DNA sequence analysis. Lenti-CRISPR virions were generated in HEK293-FT packaging cell line (R70007, Thermo Fisher Scientific) by transfecting the psPAX2 packaging plasmid (Addgene plasmid #12260), the pMD2.G plasmid (Addgene plasmid #12259) and either the lentiCRISPRv2-TRIM29, lentiCRISPRv2-STING, or lentiCRISPRv2 viral vector plasmids. Viral supernatants were harvested after 72 h. BEAS-2B or CNE1 cells were infected with lentiviral media in presence of polybrene (4 mg/ml) by spin infection (920 g at 25 °C) for 45 min. After 24 h of infection, transduced cells were selected with 1 ug/ml puromycin for 5 days. Cells were lysed for verification of TRIM29 or STING expression by immunoblotting.

### RNA and EBV DNA extraction and real-time PCR

Total cellular RNA was extracted from cultured cells using the TRIzol reagent (Invitrogen) according to the manufacturer’s instructions. cDNA was synthesized from 2 μg of the total RNA using a reverse transcriptase kit (Invitrogen). The mRNA level was evaluated by qRT-PCR using the Power SYBR Green qPCR SuperMix-UDG (Invitrogen) and was analysed on Roche Lightcycler 480. All gene expressions were normalized to the housekeeping gene GAPDH, used as an internal standard. The following primers were used: EBV-BamHI-W forward, 5′-CCCAACACTCCACCACACC-3′ and reverse, 5′-TCTTAGGAGCTGTCCGAGGG-3′; GAPDH forward, 5′-CCCCACACACATGCACTTACC-3′ and reverse, 5′-CCTAGTCCCAGGGCTTTGATT-3′. EBV DNA was extracted from EBV-infected cells using Omega tissue DNA Mini Kit (Omega) as recommended by the manufacturer. The copy number of EBV bound to the cell surface or internalized into cells was measured using TaqMan real-time PCR for detection of the BamHI-W fragment region of the EBV genome. Real-time PCR for the GAPDH DNA was used for cell-counting estimation. A calibration curve was performed with each analysis, using DNA extracted from the EBV-positive cell line Namalwa (ATCC CRL-1432), which contains two integrated viral genomes per cell, as a standard. The EBV copy number was expressed as a ratio of the copy number of the EBV genome to the copy number of the GAPDH DNA.

### Viruses and infection

HSV-1 WT strain (ATCC VR-733) was propagated and titered by plaque assay on Vero cells (ATCC CCL-81) as described previously^59, 61–64^, and human adenovirus was kept as described previously^59^. Six-week-old to eight-week-old female mice of different genotypes were infected with HSV-1 (2 × 10^7^ plaque forming units (PFU) per mouse) via tail vein injection. The viability of the infected mice was monitored for 14 days. Sera were collected at different time points to measure the productions of IFN-α, IFN-β, IL-6, and TNF-α by ELISA. Viral titers were measured by plaque assays using homogenates from brains, liver, and spleen of infected mice. For plaque assay, Vero cells were incubated with viral samples at serial dilutions for 1 h and then overlaid with 1.5% methylcellulose in MEM containing 1% FBS. Seventy-two hours later, cells were fixed in methanol and stained with 0.1% crystal violet. Plaques were counted to calculate viral titer as previously described^62–64^.

Six-week-old to eight-week-old female mice were infected intranasally with 1 × 10^8^ PFU of adenovirus in 50 µl phosphate-buffered saline (PBS) after anesthesia. Mice were killed at various time points following infection. To collect BALF from mice, tracheas were cannulated after exsanguination and lungs were washed with 1 ml of PBS. BALF samples were centrifuged (800 g, 5 min) to isolate cells and supernatants were centrifuged again (13,000 g, 1 min) to completely remove remaining cells. The concentrations of IFN-α, IFN-β, TNF-α, and IL-6 in BALF were measured by ELISA. After adenovirus infection in mice, total lung was removed and homogenized to prepare lung extracts in 1 ml of PBS (pH 7.4). The virus titers were determined by plaque assay with A549 cells (ATCC CCL-185) as previously described^65^.

### Histology

Lungs were removed from naive or infected WT and *Trim29* ^−/−^ mice and washed using PBS before being fixed with 4% formaldehyde for 1 h at 4 °C. The tissues were embedded in paraffin and processed by standard techniques. Longitudinal 5-μm sections were stained with hematoxylin and eosin.

### In vitro pull-down and immunoblot analysis

For the preparation of purified STING and TRIM29, HEK293T cells (ATCC CRL-3216) were transfected with an expression plasmids encoding full-length or truncated versions of HA- or Myc-tagged STING or TRIM29. All the above plasmids were constructed in pCMV-Myc or pCMV-HA vector (Catalog: 631604, Clontech). Lysates were prepared from the transfected cells, followed by incubation with anti-HA or anti-Myc beads. Proteins were eluted from the beads after beads were washed six times with PBS. For precipitation with anti-HA or anti-Myc beads, purified HA-tagged WT STING or truncations of STING were incubated for 2 h with purified Myc-tagged TRIM29 or purified HA-tagged TRIM29 or truncations of TRIM29 were incubated for 2 h with purified Myc-tagged STING. Beads were added; after 2 h of incubation, the bound complexes were pelleted by centrifugation. Proteins and beads were analyzed by immunoblot analysis with anti-HA or anti-Myc Abs. Uncropped scans of immunoblots are provided as Supplementary Fig. 15.

### Ubiquitination

HEK293T cells were transfected with expression plasmid encoding Myc-tagged full-length STING and with or without coexpression of HA-tagged full-length TRIM29 and its mutant T29-∆C. At 24 h after transfection, cells were treated with 25 μM MG132 for 3 h and then were collected for analysis^59, 66^. Briefly, cells were lysed and the cell lysis was heated at 65 °C for 5 min. After spinning down, the supernatants were collected and were immunoprecipitated with anti-Myc beads and then analyzed by immunoblot.

### Confocal microscopy

HEK293T cells were co-transfected with expression plasmids for HA-tagged full-length TRIM29 or its mutant T29-∆C and Myc-tagged STING. After 24 h, cells were then fixed in 4% paraformaldehyde and permeabilized with 0.1% triton-100, and then blocked for 30 min with 5% bovine serum albumin (BSA), incubated overnight at 4 °C with Alexa Fluor 555-anti-Myc and Alexa Fluor 488-anti-HA and then examined with confocal microscopy as described in our previous studies^59, 60^. BEAS-2B cells were without stimulation or stimulated with dsDNA HSV-60 for 4 h. Cells were then fixed in 4% paraformaldehyde and permeabilized with 0.1% triton-100, and then blocked for 30 min with 5% BSA, incubated with Rabbit anti-STING polyclonal antibody (1:500; Cat: 19851-1-AP, Proteintech) and mouse anti-TRIM29 monoclonal antibody (1:500; sc-166707, Santa Cruz) for 2 h, followed by Alexa Fluor 488 goat anti-rabbit secondary antibody and Alexa Fluor 594 goat anti-mouse secondary antibody for 1 h and then examined with confocal microscopy. Images of “zoomed” single cells were quantified with Nikon Confocal Software.

### Luciferase reporter gene assay

Human HeLa cells (ATCC, CCL-2) were seeded on 48-well plates (1 × 10^5^ cells per well), and then transfected with reporter vectors for *Ifnb*-firefly luciferase (100 ng) and renilla luciferase (1 ng) plus expression vector for WT STING or STING mutants (100 ng) with or without expression vector for TRIM29 (300 ng). Empty control vector was added so that a total of 550 ng of vector DNA was transfected into each well of cells. At 24 h after transfection, cells were stimulated with 25 μg/ml dsDNA from HSV I (HSV-60, 2.5 μg/ml) delivered by Lipofectamine 3000. Cells were collected after 6 h of stimulation. Luciferase activity in total cell lysates was detected by Dual-Luciferase Reporter Assay.

### Immunoprecipitation–mass spectrometry assay

D2SC cells were transfected with cytosolic VACV-70 dsDNA (5 μg/ml) using Lipofectamine 3000 for 8 h. Cells were lysed with RIPA lysis buffer (sc-24948, Santa Cruz). After spinning down, supernatants were collected and immunoprecipitated by anti-TRIM29 rabbit antibody (1:500; A301-210A; Bethyl). The immunoprecipitation samples were sent out for protein sequencing by liquid chromatography–mass spectrometry at Mass Spectrometry - Proteomics Core Laboratory in Baylor College of Medicine.

### Statistical analysis

A two-tailed unpaired Student’s *t*-test was used for statistical analysis with Microsoft Excel and GraphPad Prism Software. *P* values of less than 0.05 were considered significant unless specifically indicated.

### Data availability

The data supporting the findings of this study are available from the corresponding author upon request.

## Electronic supplementary material

Supplementary Information

## References

[1] Holgate ST (2011). The sentinel role of the airway epithelium in asthma pathogenesis. Immunol. Rev..

[2] Proud D, Leigh R (2011). Epithelial cells and airway diseases. Immunol. Rev..

[3] Weitnauer M, Mijosek V, Dalpke AH (2016). Control of local immunity by airway epithelial cells. Mucosal Immunol..

[4] Mayer AK (2007). Differential recognition of TLR-dependent microbial ligands in human bronchial epithelial cells. J. Immunol..

[5] Liu P (2007). Retinoic acid-inducible gene I mediates early antiviral response and Toll-like receptor 3 expression in respiratory syncytial virus-infected airway epithelial cells. J. Virol..

[6] Heyl KA (2014). Dectin-1 is expressed in human lung and mediates the proinflammatory immune response to nontypeable *Haemophilus influenzae*. mBio.

[7] Sun L, Wu J, Du F, Chen X, Chen ZJ (2013). Cyclic GMP-AMP synthase is a cytosolic DNA sensor that activates the type I interferon pathway. Science.

[8] Lambrecht BN, Hammad H (2012). The airway epithelium in asthma. Nat. Med..

[9] Gajewski TF, Corrales L (2015). New perspectives on type I IFNs in cancer. Cytokine Growth Factor Rev..

[10] Barber GN (2014). STING-dependent cytosolic DNA sensing pathways. Trends Immunol..

[11] Woo SR (2014). STING-dependent cytosolic DNA-sensing mediates innate immune recognition of immunogenic tumors. Immunity.

[12] Corrales L (2015). Direct activation of STING in the tumor microenvironment leads to potent and systemic tumor regression and immunity. Cell Rep..

[13] Ohkuri T (2014). STING contributes to antiglioma immunity via triggering type I IFN signals in the tumor microenvironment. Cancer Immunol. Res..

[14] Baird JR (2016). Radiotherapy combined with novel STING-targeting oligonucleotides results in regression of established tumors. Cancer Res..

[15] Lara PN (2011). Randomized phase III placebo-controlled trial of carboplatin and paclitaxel with or without the vascular disrupting agent vadimezan (ASA404) in advanced non-small-cell lung cancer. Clin. Oncol..

[16] Fu J (2015). STING agonist formulated cancer vaccines can cure established tumors resistant to PD-1 blockade. Sci. Transl. Med..

[17] Young LS, Rickinson AB (2004). Epstein-Barr virus: 40 years on. Nat. Rev. Cancer.

[18] Westphalen K (2014). Sessile alveolar macrophages communicate with alveolar epithelium to modulate immunity. Nature.

[19] Snelgrove RJ (2008). A critical function for CD200 in lung immune homeostasis and the severity of influenza infection. Nat. Immunol..

[20] Paine R (2000). Granulocyte-macrophage colony-stimulating factor in the innate immune response to *Pneumocystis carinii* pneumonia in mice. J. Immunol..

[21] Ting JP, Kastner DL, Hoffman HM (2006). CATERPILLERs, pyrin and hereditary immunological disorders. Nat. Rev. Immunol..

[22] Rathinam VA, Fitzgerald KA (2011). Innate immune sensing of DNA viruses. Virology.

[23] Shu HB, Wang YY (2014). Adding to the STING. Immunity..

[24] McLaughlin-Drubin ME, Munger K (2008). Viruses associated with human cancer. Biochim. Biophys. Acta.

[25] de Martel C (2012). Global burden of cancers attributable to infections in 2008: a review and synthetic analysis. Lancet Oncol..

[26] Cohen JI, Fauci AS, Varmus H, Nabel GJ (2011). Epstein-Barr virus: an important vaccine target for cancer prevention. Sci. Transl. Med..

[27] Hawthorn L, Stein L, Panzarella J, Loewen GM, Baumann H (2006). Characterization of cell-type specific profiles in tissues and isolated cells from squamous cell carcinomas of the lung. Lung Cancer.

[28] Dyrskjot L (2004). Gene expression in the urinary bladder: a common carcinoma in situ gene expression signature exists disregarding histopathological classification. Cancer Res..

[29] Glebov OK (2006). Gene expression patterns distinguish colonoscopically isolated human aberrant crypt foci from normal colonic mucosa. Cancer Epidemiol. Biomark. Prev..

[30] Santin AD (2004). Gene expression profiles in primary ovarian serous papillary tumors and normal ovarian epithelium: identification of candidate molecular markers for ovarian cancer diagnosis and therapy. Int. J. Cancer.

[31] Mutter GL (2001). Global expression changes of constitutive and hormonally regulated genes during endometrial neoplastic transformation. Gynecol. Oncol..

[32] Zhan F (2002). Global gene expression profiling of multiple myeloma, monoclonal gammopathy of undetermined significance, and normal bone marrow plasma cells. Blood.

[33] Zhou XM (2016). Upregulated TRIM29 promotes proliferation and metastasis of nasopharyngeal carcinoma via PTEN/AKT/mTOR signal pathway. Oncotarget.

[34] Chen Z (2016). Identification of nasopharyngeal carcinoma metastasis-related biomarkers by iTRAQ combined with 2D-LC-MS/MS. Oncotarget.

[35] Rajsbaum R, Garcia-Sastre A, Versteeg GA (2014). TRIMmunity: the roles of the TRIM E3-ubiquitin ligase family in innate antiviral immunity. J. Mol. Biol..

[36] Liu X (2012). Antitumor effects of interferon-alpha on cell growth and metastasis in human nasopharyngeal carcinoma. Curr. Cancer Drug Targets.

[37] Xing J (2016). Identification of a role for TRIM29 in the control of innate immunity in the respiratory tract. Nat. Immunol..

[38] Chen ZJ, Sun LJ (2009). Nonproteolytic functions of ubiquitin in cell signaling. Mol. Cell.

[39] Hussell T, Bell TJ (2014). Alveolar macrophages: plasticity in a tissue-specific context. Nat. Rev. Immunol..

[40] Corrales L, McWhirter SM, Dubensky TW, Gajewski TF (2016). The host STING pathway at the interface of cancer and immunity. J. Clin. Invest..

[41] Zhong B (2009). The ubiquitin ligase RNF5 regulates antiviral responses by mediating degradation of the adaptor protein MITA. Immunity.

[42] Wang Y (2015). TRIM30alpha Is a negative-feedback regulator of the intracellular DNA and DNA virus-triggered response by targeting STING. PLoS Pathog..

[43] Shi M (2008). TRIM30 alpha negatively regulates TLR-mediated NF-kappa B activation by targeting TAB2 and TAB3 for degradation. Nat. Immunol..

[44] Tsuchida T (2010). The ubiquitin ligase TRIM56 regulates innate immune responses to intracellular double-stranded DNA. Immunity.

[45] Zhang J, Hu MM, Wang YY, Shu HB (2012). TRIM32 protein modulates type I interferon induction and cellular antiviral response by targeting MITA/STING protein for K63-linked ubiquitination. J. Biol. Chem..

[46] Wang Q (2014). The E3 ubiquitin ligase AMFR and INSIG1 bridge the activation of TBK1 kinase by modifying the adaptor STING. Immunity.

[47] Hu MM (2016). Sumoylation promotes the stability of the DNA sensor cGAS and the adaptor STING to regulate the kinetics of response to DNA virus. Immunity.

[48] Konno H, Konno K, Barber GN (2013). Cyclic dinucleotides trigger ULK1 (ATG1) phosphorylation of STING to prevent sustained innate immune signaling. Cell.

[49] Tanaka Y, Chen ZJ (2012). STING specifies IRF3 phosphorylation by TBK1 in the cytosolic DNA signaling pathway. Sci. Signal.

[50] Liu S (2015). Phosphorylation of innate immune adaptor proteins MAVS, STING, and TRIF induces IRF3 activation. Science.

[51] Zhang L (2014). NLRC3, a member of the NLR family of proteins, is a negative regulator of innate immune signaling induced by the DNA sensor STING. Immunity.

[52] Zhou Q (2014). The ER-associated protein ZDHHC1 is a positive regulator of DNA virus-triggered, MITA/STING-dependent innate immune signaling. Cell Host Microbe.

[53] Luo WW (2016). iRhom2 is essential for innate immunity to DNA viruses by mediating trafficking and stability of the adaptor STING. Nat. Immunol..

[54] Medzhitov R (2007). Recognition of microorganisms and activation of the immune response. Nature.

[55] Xing J, Ly H, Liang Y (2015). The Z proteins of pathogenic but not nonpathogenic arenaviruses inhibit RIG-I-like receptor-dependent interferon production. J. Virol..

[56] Xing J, Chai Z, Ly H, Liang Y (2015). Differential inhibition of macrophage activation by lymphocytic choriomeningitis virus and pichinde virus is mediated by the Z Protein N-terminal domain. J. Virol..

[57] Tsao SW (2002). Establishment of two immortalized nasopharyngeal epithelial cell lines using SV40 large T and HPV16E6/E7 viral oncogenes. Biochim. Biophys. Acta.

[58] Zeng Y (1978). Establishment of an epitheloid cell line and a fusiform cell line from a patient with nasopharyngeal carcinoma. Sci. Sin..

[59] Zhang Z (2013). The E3 ubiquitin ligase TRIM21 negatively regulates the innate immune response to intracellular double-stranded DNA. Nat. Immunol..

[60] Zhang Z, . (2011). The helicase DDX41 senses intracellular DNA mediated by the adaptor STING in dendritic cells. Nat. Immunol..

[61] Fernandez S, Jose P, Avdiushko MG, Kaplan AM, Cohen DA (2004). Inhibition of IL-10 receptor function in alveolar macrophages by Toll-like receptor agonists. J. Immunol..

[62] Xing J, . (2013). Herpes simplex virus 1-encoded tegument protein VP16 abrogates the production of beta interferon (IFN) by inhibiting NF-kappaB activation and blocking IFN regulatory factor 3 to recruit its coactivator CBP. J. Virol..

[63] Xing J (2011). Comprehensive characterization of interaction complexes of herpes simplex virus type 1 ICP22, UL3, UL4, and UL20.5. J. Virol..

[64] Xing J, Wang S, Lin R, Mossman KL, Zheng C (2012). Herpes simplex virus 1 tegument protein US11 downmodulates the RLR signaling pathway via direct interaction with RIG-I and MDA-5. J. Virol..

[65] Subramanian T, Vijayalingam S, Chinnadurai G (2006). Genetic identification of adenovirus type 5 genes that influence viral spread. J. Virol..

[66] Weng L (2014). The E3 ubiquitin ligase tripartite motif 33 is essential for cytosolic RNA-induced NLRP3 inflammasome activation. J. Immunol..

