# Supplementary material for: TRIM29 promotes DNA virus infections by inhibiting innate immune response
Source: Nat Commun. 2017 Oct 16;8:945. doi: 10.1038/s41467-017-00101-w (PMC5643338; doi:10.1038/s41467-017-00101-w)
Supplement: Supplementary file 1 — Supplementary Information [file 41467_2017_101_MOESM1_ESM.pdf]

File name: Supplementary Information
Description: Supplementary Figures and Supplementary Tables

Supplementary Table 1. STING is in the TRIM29-binding protein complex in D2SC cells

NCBI gi no.	Protein name	Hits
6680674	RAC-beta serine/threonine-protein kinase [Mus musculus]	22
34368584	Zinc finger CCCH domain-contraning protein 15 [Mus musculus]	15
13385872	Interleukin enhancer-binding factor 2 [Mus musculus]	14
160333881	Tripartite motif-containing protein 29 [Mus musculus]	13
110835719	B-cell scaffold protein with ankyrin repeats [Mus musculus]	11
7949020	Cyclin-dependent kinase 2 isoform 2 [Mus musculus]	11
356995870	Serine/threonine-protein kinase D2 [Mus musculus]	11
18699998	Cyclin-dependent kinase 9 [Mus musculus]	9
22094081	Dual specificity mitogen-activated protein kinase3 [Mus musculus]	9
20149752	TGF-beta-activated kinase 1 and MAP3K7-binding protein 2 [Mus musculus]	8
356582268	Mitogen-activated protein kinase 4 isoform 4 [Mus musculus]	7
165905605	3'-5' exoribonuclease 1 [Mus musculus]	5
18497290	RAF proto-oncogene serine/threonine-protein kinase [Mus musculus]	4
114326482	Signal transducer and activator of transcription 1 isoform 2 [Mus musculus]	4
145587104	X-ray repair cross-complementing protein 6 [Mus musculus]	4
115495455	5'-3' exoribonuclease 1 [Mus musculus]	3
6754632	Mitogen-activated protein kinase 1 [Mus musculus]	3
40254249	Nuclear factor related to kappa-B-binding protein [Mus musculus]	3
254692993	Transmembrane protein 173 [Mus musculus]	3
315013583	Death domain-associated protein 6 [Mus musculus]	2
6678794	Dual specificity mitogen-activated protein kinase 1 [Mus musculus]	2

D2SC cells were treated with cytosolic VACV-70 dsDNA for 8h. Cells lysates were prepared, followed by anti-TRIM29 immunoprecipitation and protein sequencing by liquid chromatography-mass spectrometry. NCBI gi no: unique protein identification number; Hits: the number of peptides ions matched that associated protein.

Supplementary Table 2. The possible ubiquitination sites of STING

<table>
<tr><th colspan="4">STING Ubiquitination Sites</th></tr>
<tr><td colspan="4">MPHSSLHPSIPCPRGHGAQKAALVLLSACLVTLWGLGEPPEHTLRYLVLHLASLQLGLLLNGVCSLAEEL
RHIHSRYRGSYWRTVRACLGCPLRRGALLLLSIYFYYSLPNAVGPPFTWMLALLGLSQALNILLGLKGLAP
AEISAVCEKGNFNVAHGLAWSYYIGYLRLILPELQARIRTYNQHYNNLLRGAVSQRLYILLPLDCGVPDNLS
MADPNIRFLDKLPQQTGDHAGIKDRVYSNSIYELLENGQRAGTCVLEYATPLQTLFAMSQYSQAGFSRED
RLEQAKLFCRTLEDILADAPESQNNCRLIAYQEPADDSSFSLSQEVLRHLRQEEKEEVTVGSLKTSAVPST
STMSQEPELLISGMEKPLPLRTDFS</td></tr>
<tr><th>Residue</th><th>Score</th><th colspan="2">Ubiquitinated</th></tr>
<tr><td>20</td><td>0.42</td><td colspan="2">No</td></tr>
<tr><td>137</td><td>0.54</td><td colspan="2">No</td></tr>
<tr><td>150</td><td>0.61</td><td colspan="2">No</td></tr>
<tr><td>224</td><td>0.36</td><td colspan="2">No</td></tr>
<tr><td>236</td><td>0.52</td><td colspan="2">No</td></tr>
<tr><td>289</td><td>0.30</td><td colspan="2">No</td></tr>
<tr><td>338</td><td>0.79</td><td colspan="2">Yes Medium confidence</td></tr>
<tr><td>347</td><td>0.87</td><td colspan="2">Yes High confidence</td></tr>
<tr><td>370</td><td>0.86</td><td colspan="2">Yes High confidence</td></tr>
<tr><th>Label</th><th>Score range</th><th>Sensitivity</th><th>Specificity</th></tr>
<tr><td>Low confidence</td><td>0.62 ≤ s ≤ 0.69</td><td>0.464</td><td>0.903</td></tr>
<tr><td>Medium confidence</td><td>0.69 ≤ s ≤ 0.84</td><td>0.346</td><td>0.950</td></tr>
<tr><td>High confidence</td><td>0.84 ≤ s ≤ 1.00</td><td>0.197</td><td>0.989</td></tr>
</table>

The possibility of unbiquitination of lysine residues in STING (middle panel) was predicted from the amino acid sequence of STING (top panel) by “UbPred: predictor of protein ubiquitination sites”.

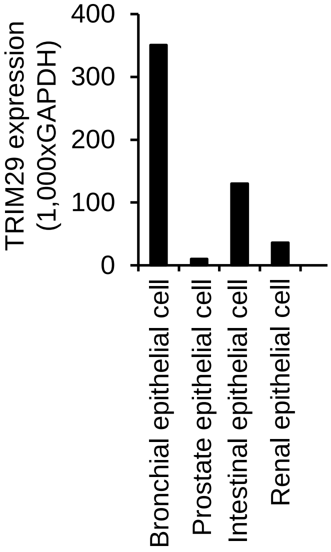

Supplementary Figure 1. The expression of TRIM29 in different human epithelial cells.

Total RNA was isolated from human bronchial epithelial cells, prostate epithelial cells, intestinal epithelial cells and renal epithelial cells and subjected to real time PCR. The profile of TRIM29 expression in different cells is indicated.

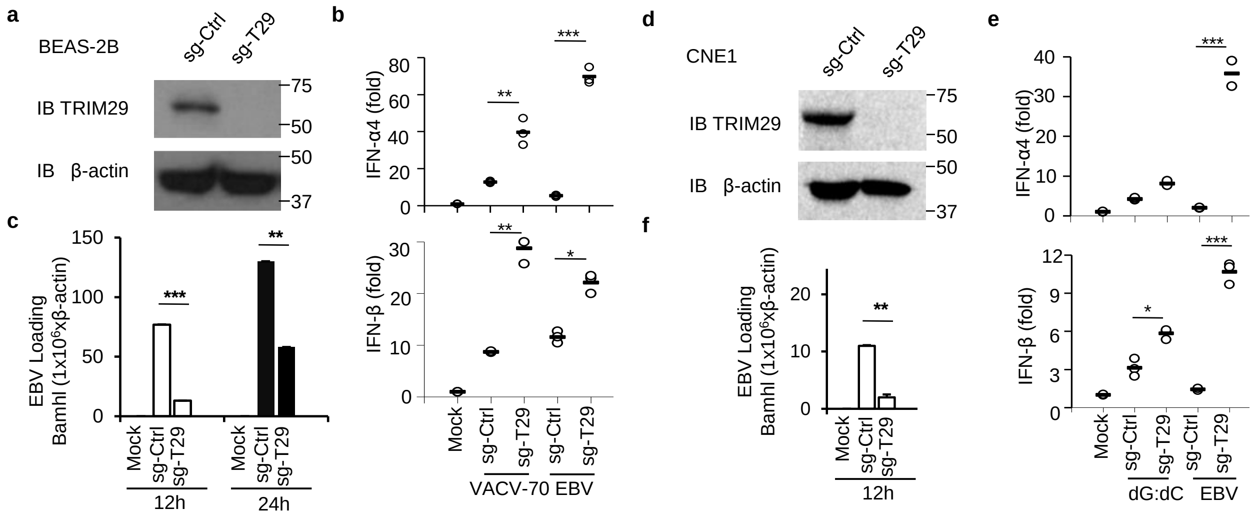

Supplementary Figure 2. TRIM29 negative regulate the production of IFN-I in BEAS-2B and CNE1 cells in response to cytosolic dsDNA or EBV infection.

(**a,d**) Immunoblot analysis of TRIM29 in human bronchial epithelial BEAS-2B cells (**a**) or nasopharyngeal carcinoma CNE1 cells treated with CRISPR vector control (sg-Ctrl) or sgRNA targeting 5'UTR region of TRIM29 (sg-T29) using CRISPR/cas9 technology. The β-actin serves as a loading control throughout. (**b,e**) Quantification of IFN-α4 and IFN-β mRNA expression in BEAS-2B (**b**) or CNE1 cells (**e**) treated with CRISPR vector control (sg-Ctrl) or sgRNA targeting 5'UTR region of TRIM29 (sg-T29) and then stimulated for 6h with dsDNA VACV-70 (5 μg/ml) or poly(dG:dC) (dG:dC, 5 μg/ml) delivered by Lipofectamine 3000 or infection with EBV. Virus was used at a multiplicity of infection (MOI) of 5. (**c,f**) Quantification of EBV DNA loading in BEAS-2B cells (**c**) or CNE1 cells (**f**) treated with CRISPR vector control (sg-Ctrl) or sgRNA targeting 5'UTR region of TRIM29 (sg-T29) and left unstimulated (Mock) and then infected with EBV for 12h or 24h. Individual circles represent the value from each independent experiment; small horizontal lines indicate the average of triplicates. *P<0.05, **P<0.001, ***P<0.0001 (unpaired t test). Mock, cells without stimulation or infection. The position of protein markers (shown in kDa) is indicated on the right. Data are representative of three independent experiments.

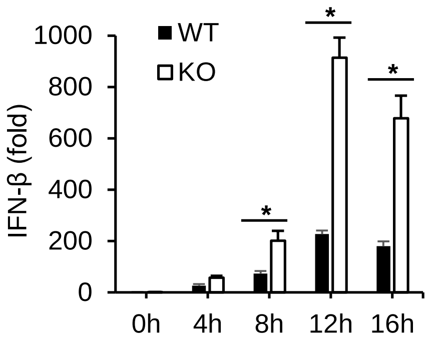

Supplementary Figure 3. The kinetics of IFN-β expression in TRIM29 WT and KO BMDCs stimulated by dsDNA.

Quantification kinetics of IFN-β mRNA expression in TRIM29 WT and KO BMDCs stimulated for 4h, 8h, 12h and 16h with dsDNA from vaccinia virus (VACV-70, 2.5 μg/ml) delivered by Lipofectamine 3000. *P<0.0001 (unpaired t test). Data are representative of two independent experiments.

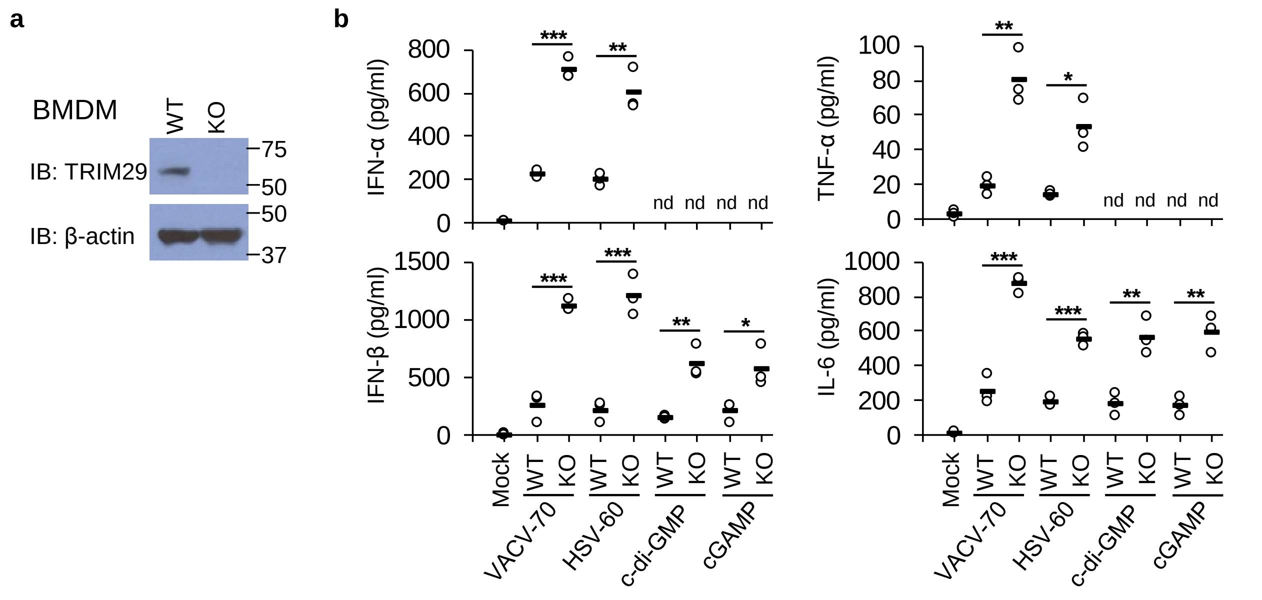

Supplementary Figure 4. TRIM29 plays an important role in sensing viral dsDNA, c-di-GMP and cGAMP in BMDMs.

(**a**) Immunoblot analysis of TRIM29 (top blot) and β-actin (bottom blot) in BMDMs isolated from wild-type (WT) and *Trim29*$^{-/-}$ (KO) mice. The position of protein markers (shown in kDa) is indicated on the right. (**b**) ELISA of IFN-α, IFN-β, TNF-α and IL-6 in BMDMs from wild-type (WT) and *Trim29*$^{-/-}$(KO) mice after 16 h of stimulation with dsDNA from vaccinia virus (VACV-70, 2.5 μg/ml), dsDNA from HSV-1 virus (HSV-60, 2.5 μg/ml), c-di-GMP (2.5 μg/ml) or cGAMP (1.0 μg/ml) delivered by Lipofectamine 3000. Each symbol represents an independent experiment; small horizontal lines indicate the average of triplicates. **P* < 0.05, ***P* < 0.01 and ****P* < 0.001 (unpaired *t* test). The “nd” is defined as not detectable. Data are representative of three independent experiments.

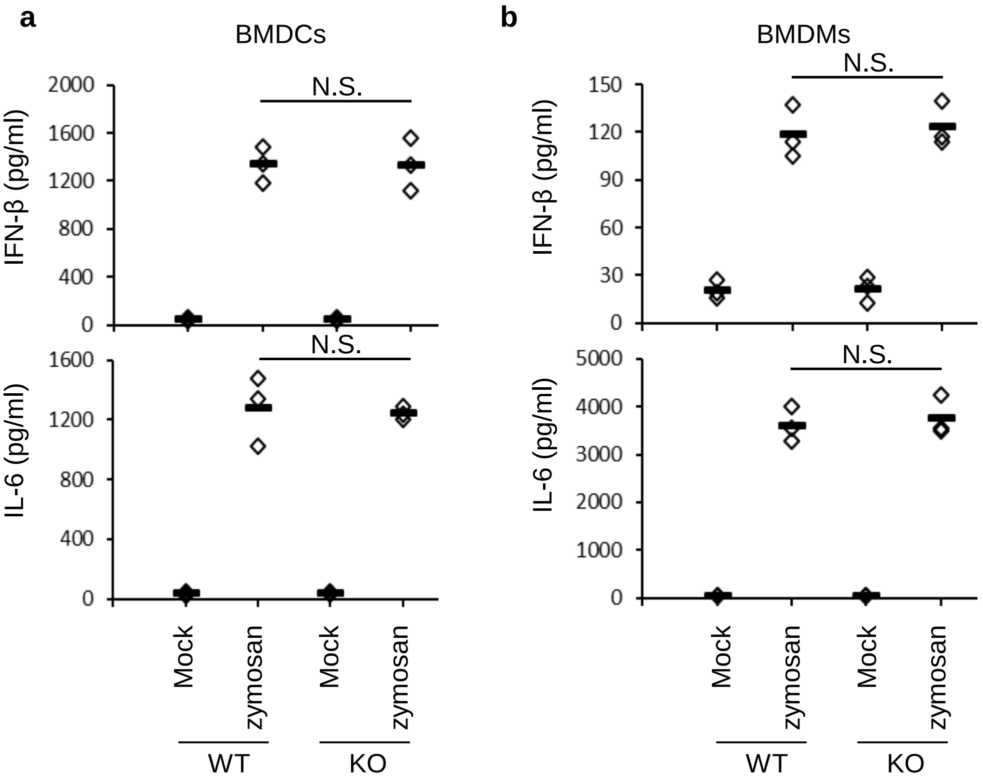

Supplementary Figure 5. *Trim29*-knockout cells have normal IFN-β and IL-6 production in response to zymosan.

(**a**) ELISA of IFN-β and IL-6 in BMDCs from wild-type (WT) and *Trim29*$^{-/-}$(KO) mice after 16 h of stimulation with zymosan (20 μg/ml). (**b**) ELISA of IFN-β and IL-6 in BMDMs from WT and KO mice after 16 h of stimulation with zymosan (20 μg/ml). Each symbol represents an independent experiment; small horizontal lines indicate the average of triplicates. N.S., not significant (unpaired *t* test). Data are representative of three independent experiments.

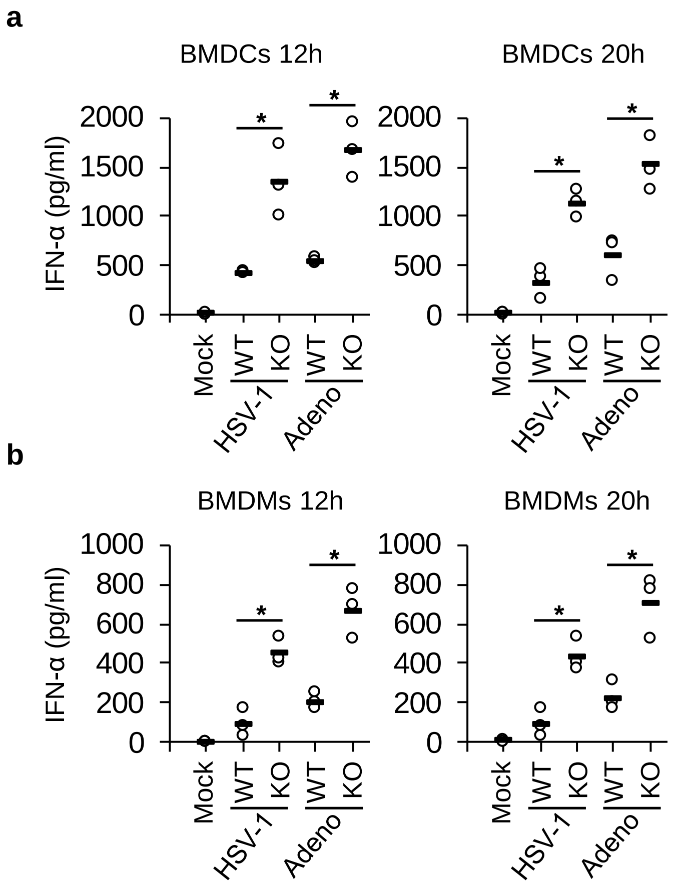

Supplementary Figure 6. TRIM29 negatively regulates IFN-α production in BMDCs and BMDMs upon DNA viral infection.

(**a**) ELISA of IFN-α in BMDCs from wild-type (WT) and *Trim29*$^{-/-}$ (KO) mice at 12h or 20h of infection without (Mock) or with HSV-1 or adenovirus (Adeno) infection. (**b**) ELISA of IFN-α in BMDMs from WT and KO mice at 12h or 20h of infection without (Mock) or with HSV-1 or adenovirus (Adeno) infection. Each symbol represents an independent experiment; small horizontal lines indicate the average of triplicates. *$P < 0.01$ (unpaired *t* test). Data are representative of three independent experiments.

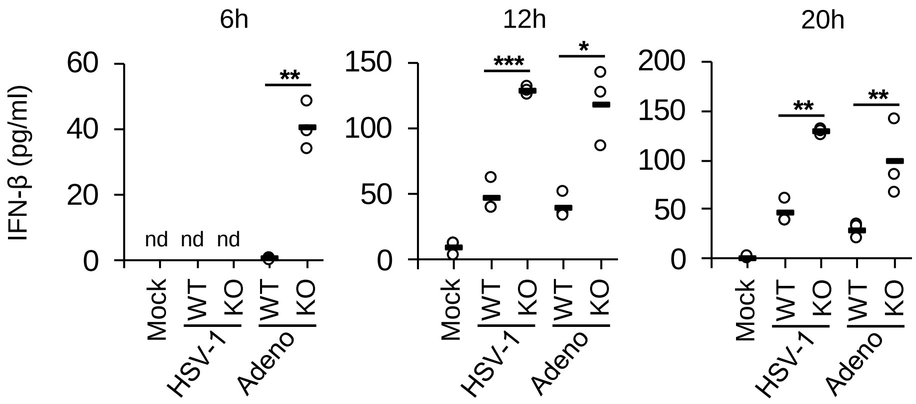

Supplementary Figure 7. TRIM29 negatively regulates IFN-β production in BMDMs upon DNA viral infection.

ELISA of IFN-β in BMDMs from wild-type (WT) and *Trim29*$^{-/-}$(KO) mice mock infected or infected with HSV-1 or Adenovirus (Adeno) at MOI of 5 for 6h, 12h or 20h. Each symbol represents an independent experiment; small horizontal lines indicate the average of triplicates. *P < 0.05, **P < 0.01 and ***P < 0.001 (unpaired t test). The "nd" is defined as not detectable. Data are representative of three independent experiments.

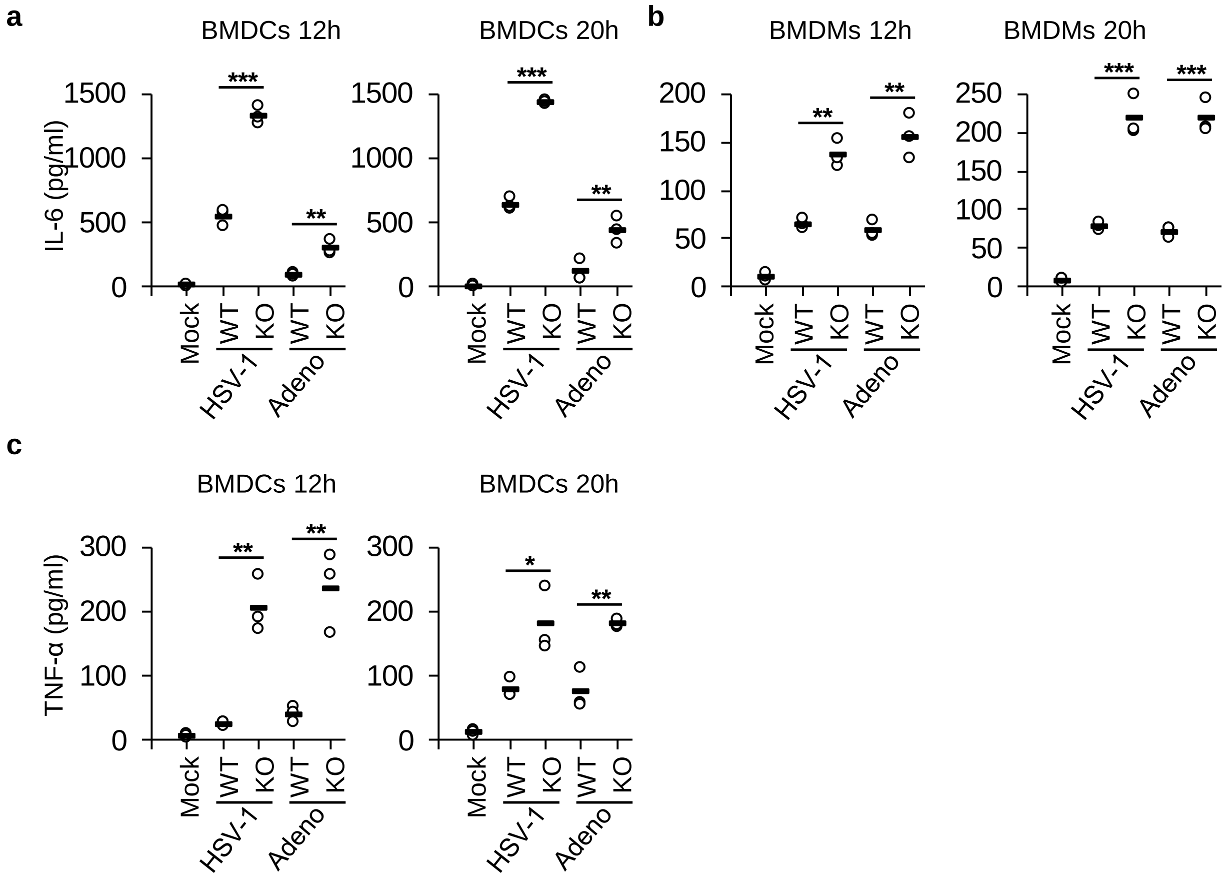

Supplementary Figure 8. TRIM29 negatively regulates the productions of TNF-α and IL-6 in BMDCs and BMDMs upon DNA viral infection.

(**a**) ELISA of IL-6 in BMDCs from wild-type (WT) and *Trim29*$^{-/-}$(KO) mice at 12h or 20h of infection without (Mock) or with HSV-1 or adenovirus (Adeno). (**b**) ELISA of IL-6 in BMDMs from WT and KO mice at 12h or 20h of infection without (Mock) or with HSV-1 or adenovirus (Adeno). (**c**) ELISA of TNF-α in BMDCs from WT and KO mice at 12h or 20h of infection without (Mock) or with HSV-1 or adenovirus (Adeno). Each symbol represents an independent experiment; small horizontal lines indicate the average of triplicates. **P* < 0.05, ***P* < 0.01 and ****P* < 0.001 (unpaired *t* test). Data are representative of three independent experiments.

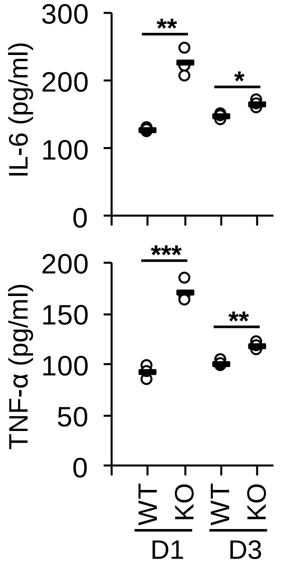

Supplementary Figure 9. TRIM29 negatively regulates IL-6 and TNF-α production in BALF upon adenovirus infection *in vivo.*

ELISA of IL-6 and TNF-α in BALF samples from wildtype mice (WT) and *Trim29*$^{-/-}$ mice (KO) at day 1 (D1) or day 3 (D3) of intranasal infection with adenovirus. Each symbol represents an independent experiment; small horizontal lines indicate the average (of triplicates). *p<0.05, ***P*<0.01, ****P*<0.001 (student's t-test). Data are representative of three independent experiments.

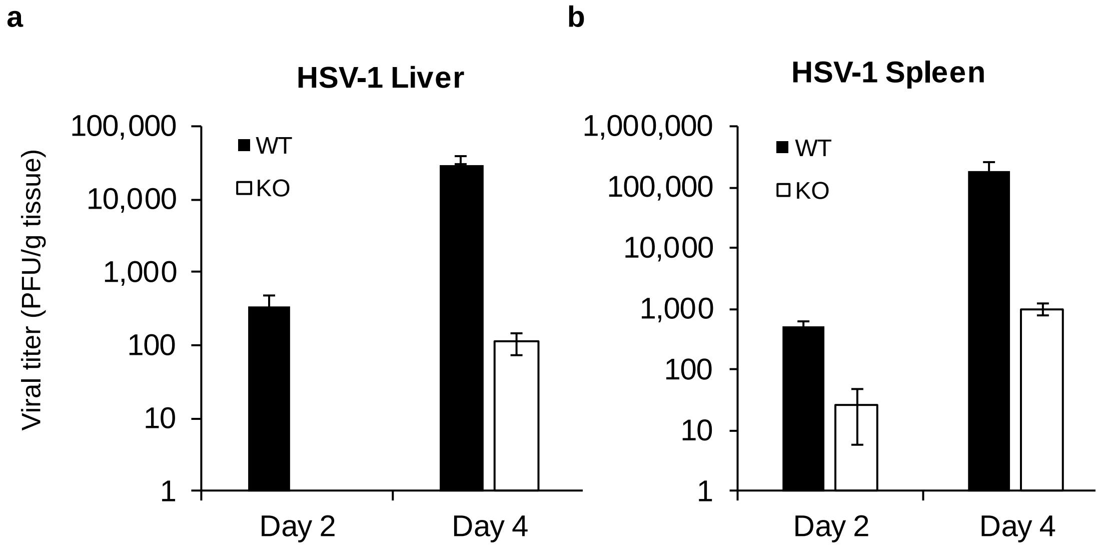

Supplementary Figure 10. Viral titers in the lung and spleen from wild-type (WT) and TRIM29$^{-/-}$ (KO) mice after intravenous injection of HSV-1.

(**a,b**) The organs liver (**a**) and spleen (**b**) from wild-type (WT) and *Trim29*$^{-/-}$ (KO) mice intravenously injected with HSV-1 virus (2×10^{7} pfu for each mouse) were homogenized on days 2 (D2) and 4 (D4), followed by viral titer examination. n=3 for each strain mice. Data are representative of three independent experiments (error bars, s.d.). Data are representative of three independent experiments.

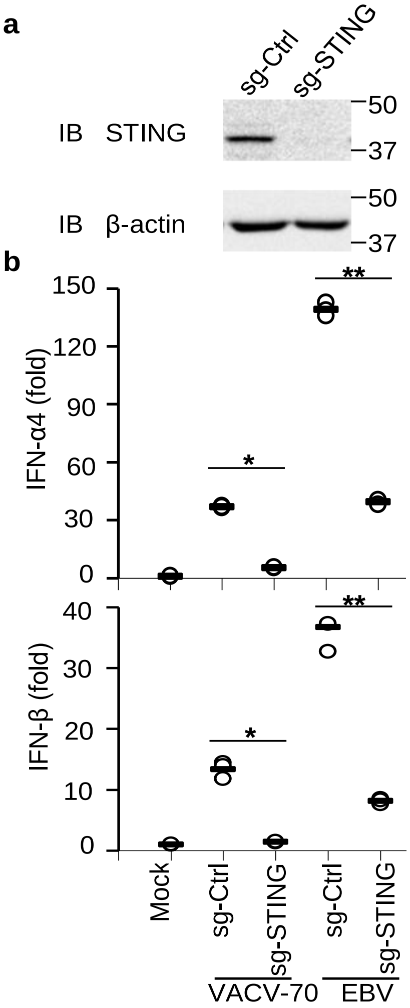

Supplementary Figure 11. STING plays critical role in anti-EBV infection in human nasopharyngeal carcinoma CNE1 cells.

(**a**) Immunoblot analysis of STING in human nasopharyngeal carcinoma CNE1 cells treated with CRISPR vector control (sg-Ctrl) or sgRNA targeting mRNA coding region of STING (sg-STING) using CRISPR/cas9 technology. The β-actin serves as a loading control. (**b**) Quantification of IFN-α4 and IFN-β mRNA expression in CNE1 cells treated with CRISPR vector control (sg-Ctrl) or sgRNA targeting mRNA coding region of STING (sg-STING) and then stimulated for 6h with dsDNA VACV-70 (5 μg/ml) or infection with EBV. Virus was used at a multiplicity of infection (MOI) of 5. Individual circles represent the value from each independent experiment; small horizontal lines indicate the average of triplicates. *P<0.001, **P<0.0001 (unpaired t test). Mock, cells without stimulation or infection. The position of protein markers (shown in kDa) is indicated on the right. Data are representative of three independent experiments.

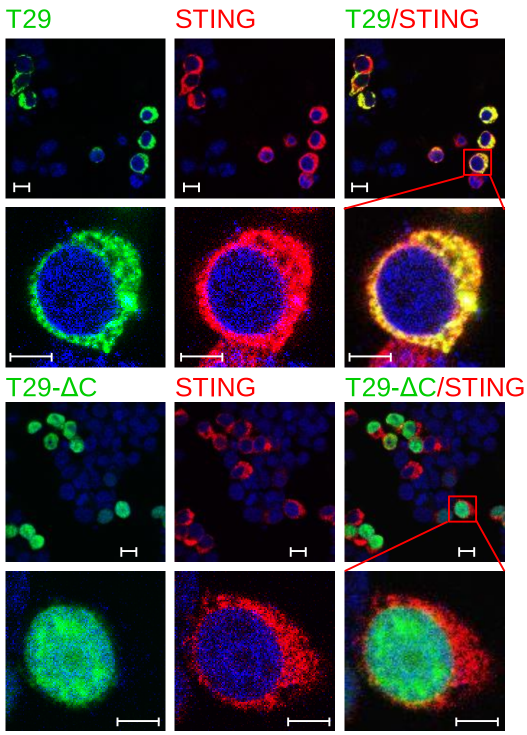

Supplementary Figure 12. Colocalization of overexpressed TRIM29 and STING in HEK293T cells.

Confocal microscopy of HEK293T cells cotransfected with expression plasmids for Myc-tagged STING (red) and HA-tagged TRIM29 or truncation T29-ΔC (losing binding site of STING, green); the DNA-intercalating dye DAPI serves as a marker of nuclei (blue). Scale bars represent 10 μm for original images and 5 μm for enlarged images. Data are representative of three independent experiments with similar results.

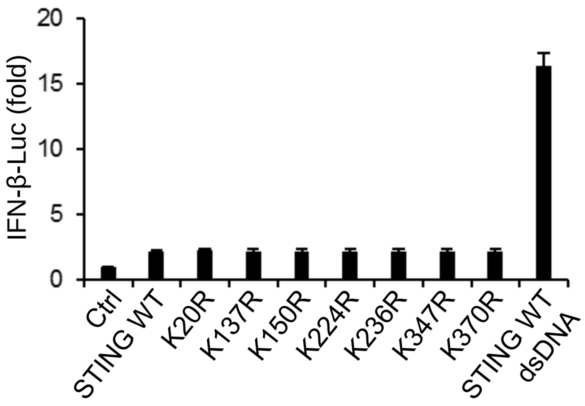

Supplementary Figure 13. The *Ifnb* promoter was activated by STING with dsDNA stimulation.

Activation of the *Ifnb* promoter in human HeLa cells transfected with an *Ifnb* luciferase reporter, plus expression vector (each 100 ng) for wild-type STING or various STING mutants alone with or without stimulation of dsDNA from vaccinia virus (2.5 μg/ml) for 8h; results are presented as fold induction compared to control (Ctrl). Data are representative of three independent experiments.

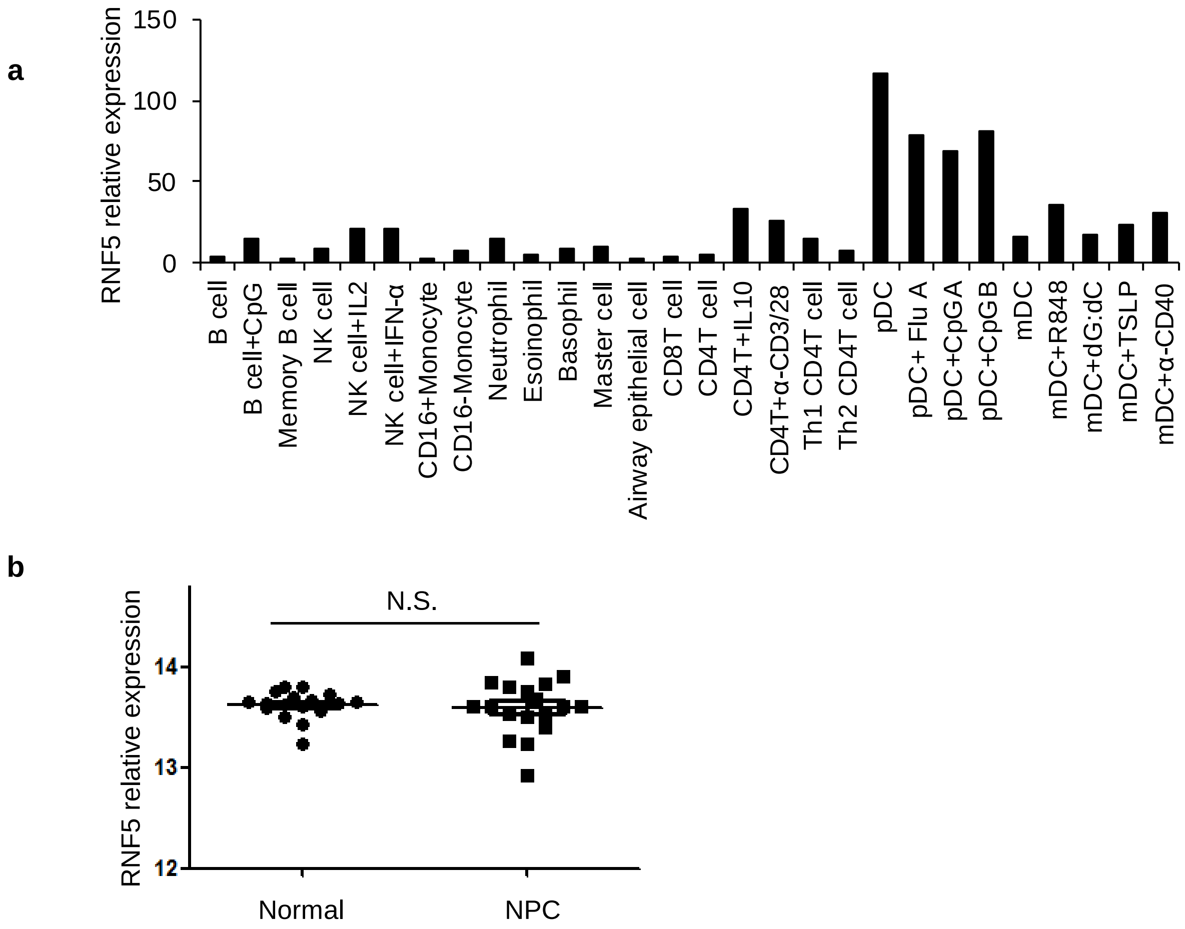

Supplementary Figure 14. The expression of RNF5 in human cells.

(**a**) Human myeloid cells and lymphoid cells were purified from peripheral blood mononuclear cells (PBMCs) using a cell sorter. Total RNA was isolated from these primary cells and primary airway epithelial cells induced or not to chip hybridization and microarray. (**b**) Total RNA was isolated from human healthy nasopharyngeal epithelial cells (Normal) or nasopharyngeal carcinoma cells (NPC) to chip hybridization and microarray. N.S., not significant (unpaired *t* test). Data are representative of two independent experiments.

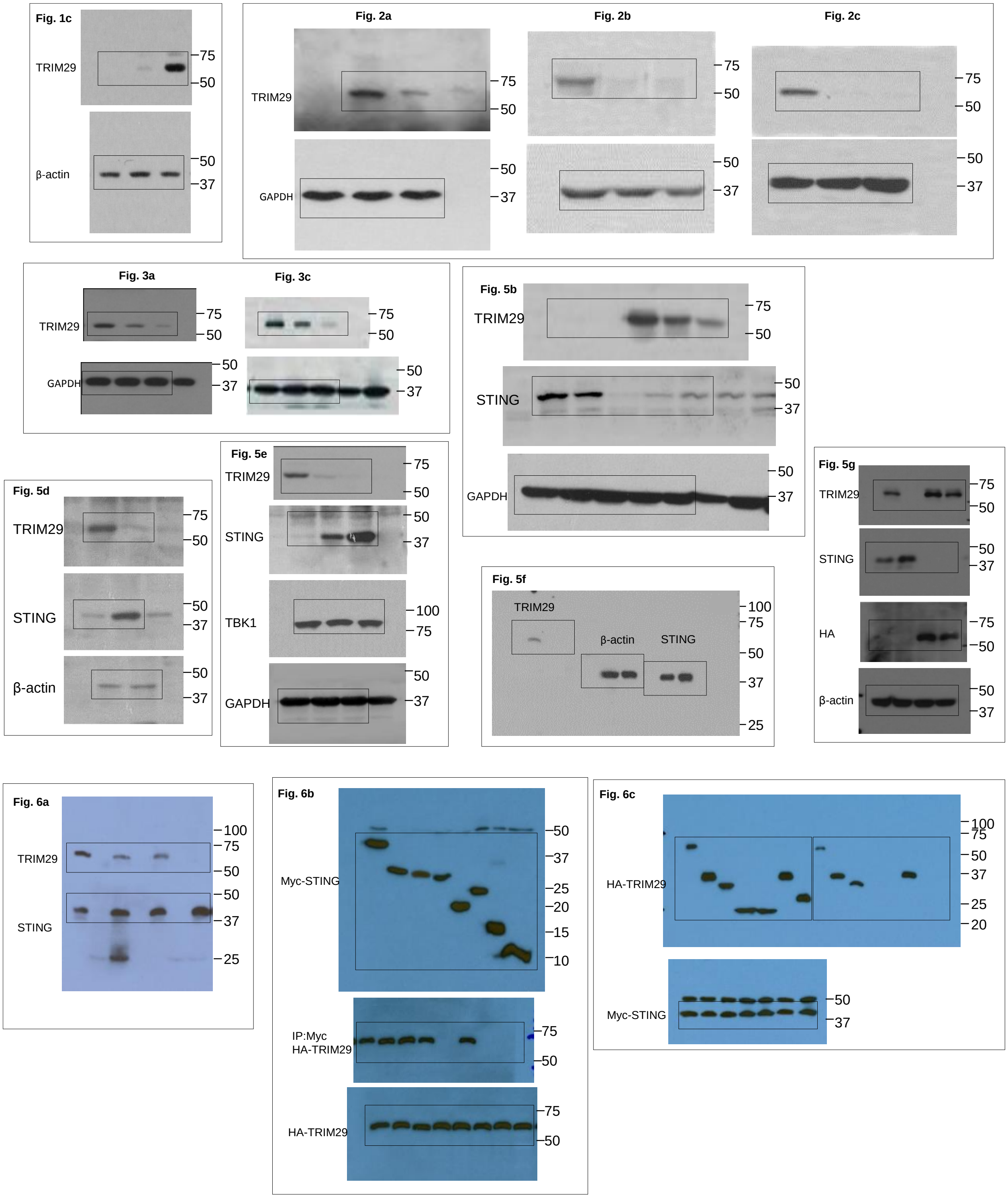

Supplementary Figure 15. Uncropped images of blots in Figure 1-3, 5 and 6.

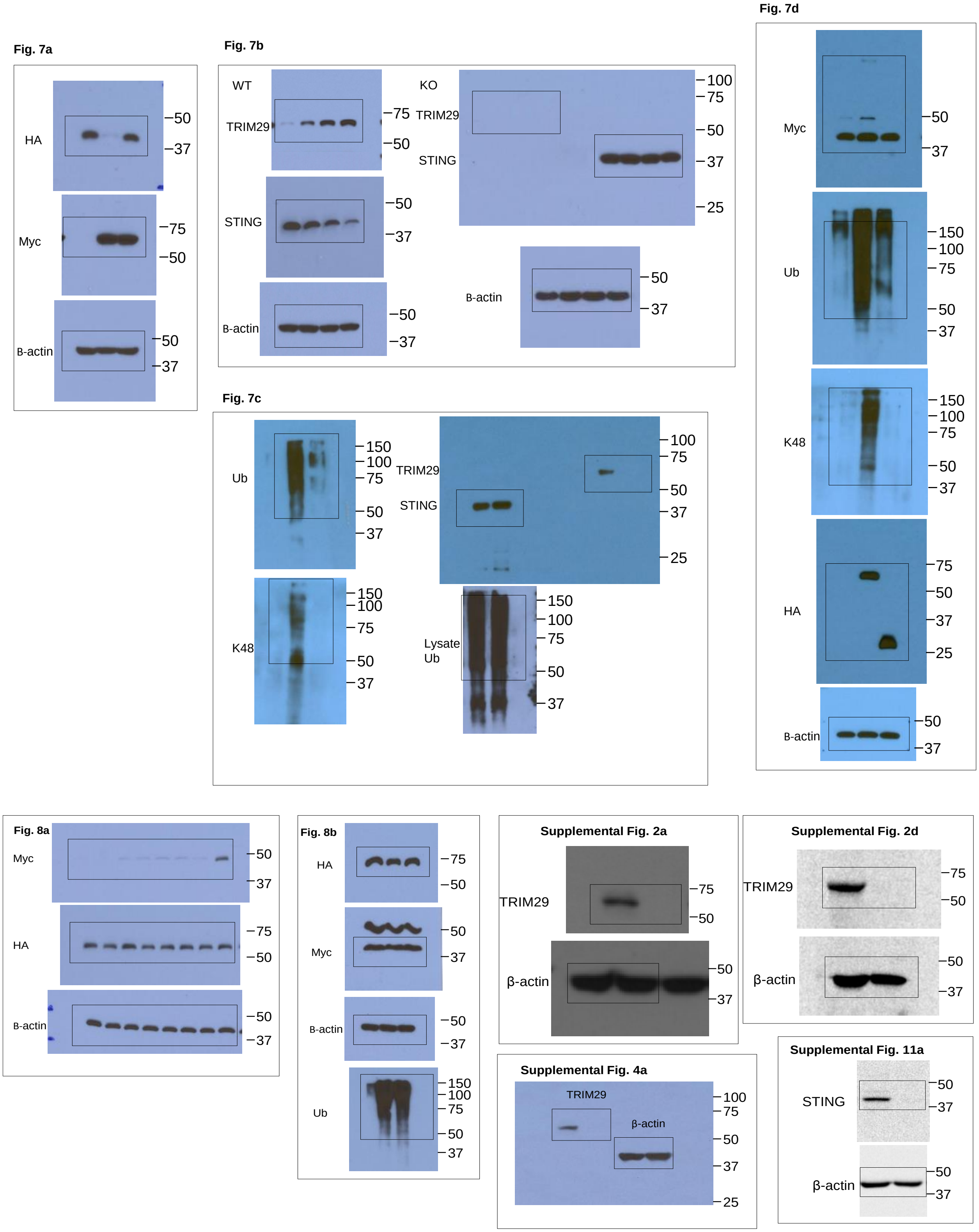

Supplementary Figure 15 (continued). Uncropped images of blots in Figure 7-8 and Supplementary Figure 2, 4 and 11.
